# Protein Identification for Stroke Progression via Mendelian Randomization in Million Veteran Program and UK Biobank

**DOI:** 10.1161/STROKEAHA.124.047103

**Published:** 2024-07-23

**Authors:** Andrew R. Elmore, Nimish Adhikari, April E. Hartley, Hugo Javier Aparicio, Daniel C. Posner, Gibran Hemani, Kate Tilling, Tom R. Gaunt, Peter W.F. Wilson, Juan P. Casas, John Michael Gaziano, George Davey Smith, Lavinia Paternoster, Kelly Cho, Gina M. Peloso

**Affiliations:** NIHR Bristol Biomedical Research Centre, University Hospitals Bristol and Weston NHS Foundation Trust and University of Bristol, United Kingdom (A.R.E., A.E.H., G.H., K.T., T.R.G., G.D.S., L.P.).; MRC Integrative Epidemiology Unit, Bristol Medical School, University of Bristol, United Kingdom (A.R.E., A.E.H., G.H., K.T., T.R.G., G.D.S., L.P.).; Veteran’s Affairs Healthcare System, Boston, MA (N.A., D.C.P., J.P.C., J.M.G., K.C., G.M.P.).; Department of Biostatistics, Boston University School of Public Health, MA (N.A., G.M.P.).; Department of Neurology, Boston University Chobanian & Avedisian School of Medicine, MA (H.J.A.).; Boston Medical Center, MA (H.J.A.).; Division of Aging, Brigham and Women’s Hospital, Harvard Medical School, Boston, MA (J.P.C., J.M.G., K.C.).; Atlanta VA Medical Center, GA (P.W.F.W.).

**Keywords:** blood proteins, genome-wide association study, inflammation, prognosis, stroke

## Abstract

**BACKGROUND:**

Individuals who have experienced a stroke, or transient ischemic attack, face a heightened risk of future cardiovascular events. Identification of genetic and molecular risk factors for subsequent cardiovascular outcomes may identify effective therapeutic targets to improve prognosis after an incident stroke.

**METHODS:**

We performed genome-wide association studies for subsequent major adverse cardiovascular events (MACE; n_cases_=51 929; n_controls_=39 980) and subsequent arterial ischemic stroke (AIS; n_cases_=45 120; n_controls_=46 789) after the first incident stroke within the Million Veteran Program and UK Biobank. We then used genetic variants associated with proteins (protein quantitative trait loci) to determine the effect of 1463 plasma protein abundances on subsequent MACE using Mendelian randomization.

**RESULTS:**

Two variants were significantly associated with subsequent cardiovascular events: rs76472767 near gene *RNF220* (odds ratio, 0.75 [95% CI, 0.64–0.85]; *P*=3.69×10^−8^) with subsequent AIS and rs13294166 near gene *LINC01492* (odds ratio, 1.52 [95% CI, 1.37–1.67]; *P*=3.77×10^−8^) with subsequent MACE. Using Mendelian randomization, we identified 2 proteins with an effect on subsequent MACE after a stroke: CCL27 ([C-C motif chemokine 27], effect odds ratio, 0.77 [95% CI, 0.66–0.88]; adjusted *P*=0.05) and TNFRSF14 ([tumor necrosis factor receptor superfamily member 14], effect odds ratio, 1.42 [95% CI, 1.24–1.60]; adjusted *P*=0.006). These proteins are not associated with incident AIS and are implicated to have a role in inflammation.

**CONCLUSIONS:**

We found evidence that 2 proteins with little effect on incident stroke appear to influence subsequent MACE after incident AIS. These associations suggest that inflammation is a contributing factor to subsequent MACE outcomes after incident AIS and highlights potential novel targets.

Stroke remains a significant public health concern worldwide. With its potential to cause profound disabilities and mortality, it necessitates continued research efforts to unravel its multifaceted etiology, identify modifiable risk factors, and develop effective therapeutic interventions.

Arterial ischemic stroke (AIS) accounts for ≈85% of all stroke cases and arises from occlusion of cerebral blood vessels, leading to inadequate perfusion and a subsequent ischemic cascade.^1^ Through the study of incident stroke events, modifiable factors such as hypertension, diabetes, dyslipidemia, atrial fibrillation, obesity, and lifestyle behaviors have been identified, which may offer promising targets for prevention.^2^ Whether targeting the same factors offers avenues for effective treatment after the incident event is unclear.

Genome-wide association studies (GWAS) are usually performed on disease status for incident events, but expanding them to subsequent events could provide us with novel biological insights about stroke progression, which may be more relevant for drug identification opportunities.^3^ GWAS of stroke incidence have previously observed 32 loci associated with stroke and stroke subtypes^4^ with a recent study adding 5 more novel loci for stroke incidence.^5^ GWAS of disease progression can provide genetic risk factors that may be independent of the incident event. Since GWAS of disease progression include only individuals with incident disease, this can lead to the statistical problem of collider bias (or index event bias), where shared confounders between incident and subsequent events can uncover spurious associations and biased estimates of effects, even among genetic risk factors.^3^

Mendelian randomization (MR) is an established statistical method that uses genetic variants to assess putative causal relationships between genetically proxied protein abundance on incident AIS and subsequent AIS and major adverse cardiovascular events (MACE).^6^ The main advantage MR has over traditional observational epidemiological methods is that MR can imply causality between an exposure and an outcome because it is less liable to common epidemiological biases, such as confounding and reverse causality. For biases that MR does not account for, sensitivity analyses can assess whether results are robust. One such method is colocalization, which is used to identify whether a genetic variant is shared by 2 traits and is a necessary condition for causality.^7^

In this study, we perform GWAS of subsequent AIS and MACE after incident AIS in the Million Veteran Program (MVP) and UK Biobank (UKB) stratified by ancestry and meta-analyzed across ancestries (Figure 1). We then use our subsequent events GWAS to perform MR for plasma protein abundances using protein quantitative trait loci (pQTLs) from UKB Pharma Proteomics Project. Our genetic study aims to mimic a stroke prevention trial where recruitment into the trial is based on having a primary stroke event.

**Figure 1.**
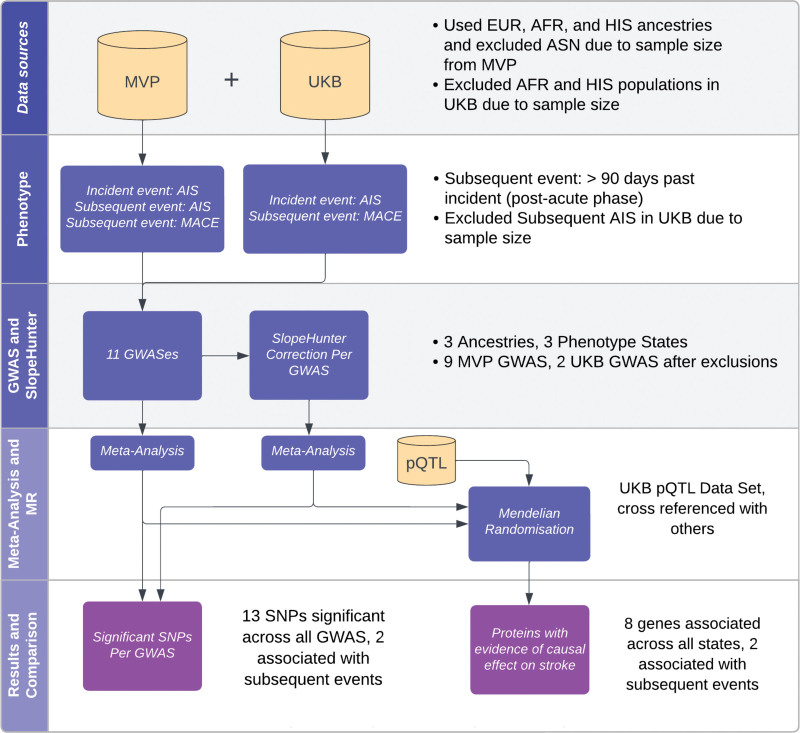
**Flowchart of the methodological processes for analyzing stroke data.** AFR indicates African ancestry; AIS, acute ischemic stroke; ASN, Asian; EUR, European; GWAS, genome-wide association study; HIS, Hispanic; MACE, major acute cardiovascular event; MR, Mendelian randomization; MVP, Million Veterans Program; pQTL, protein quantitative trait loci; SNP, single-nucleotide polymorphism; and UKB, UK Biobank. *All results compared unadjusted and Slope-Hunter adjusted results.

## METHODS

### Data Availability

GWAS and meta-analyzed results will be available under dbGaP (database of Genotypes and Phenotypes) accession phs001672.

https://www.ncbi.nlm.nih.gov/projects/gap/cgi-bin/study.cgi?study_id=phs001672

### Genome-Wide Association Studies

#### Phenotype Definitions

Incident stroke was defined as any diagnosis of AIS or transient ischemic attack using hospital-linked data (Supplemental Material).

#### UK Biobank

UKB is a prospective cohort study with over 500 000 participants 40 to 69 (average, 56.5) years of age when recruited in 2006 to 2010, and 54% of participants are women.^8^ Information on the genotype imputation, quality control, and GWAS is available in the Supplemental Material.

#### Million Veteran Program

MVP is a continually growing cohort of over 850 000 participants by 2021,^9^ 8% women, with an average age of 61.9 years.^10^ Information on the genotyping, imputation, quality control, and GWAS is available in the Supplemental Material.

#### Collider Bias Sensitivity Analysis and Correction

To perform a correction for collider bias for subsequent stroke, we standardized the data^11^ and used Slope-Hunter,^12^ a method that uses a mix of thresholding and mixed model clustering to quantify the bias and present a corrected estimate of the progression effect of a subsequent stroke (Supplemental Material).

#### Expected Versus Observed Replication

To determine whether the GWAS results of subsequent stroke are different from incident stroke, we used the approach described in the study by Okbay et al,^13^ which determines replication performance by accounting for differential power. Here, we used the approach to determine the extent to which incidence stroke GWAS hits are replicated in subsequent stroke, compared against the power-adjusted expected replication rate.

### Multiancestry Comparison and Meta-Analysis

Two meta-analyses were performed. First, European-only meta-analysis was performed across UKB and MVP. Second, a meta-analysis of all individuals, including each ancestry from MVP (European, African, and Hispanic) and Europeans from UKB was conducted using a fixed-effects model. We completed this meta-analysis for both original and Slope-Hunter adjusted results and compared the results. Both meta-analyses and heterogeneity score calculations were performed using the software METAL.^14^ The Cochran Q statistic was used to test for heterogeneity between ancestries.^14^ We limited our association results to variants with a minor allele frequency >1%, and we set our genome-wide significance threshold to 5×10^−8^.

We ran tissue expression analysis on subsequent MACE using Functional Mapping and Annotation of GWAS,^15^ including MAGMA^16^ Tissue Expression Analysis, to investigate whether there were any significant correlations between the subsequent GWAS and tissue expression.

### MR Against Protein Abundance

Using the meta-analyzed GWAS results and existing available pQTL data sets, we performed MR for each outcome with a panel of 1463 plasma proteins as potential causal risk factors. Measuring proteins at population scale could help discover novel clinical biomarkers and improve fine-mapping of causal genes linked to complex diseases.^17^ To account for multiple testing, *P* values were adjusted using false discovery rate and are subsequently reported as adjusted *P*.

pQTLs were extracted from pQTL studies from UKB Pharma Proteomics Project (54 306 participants, 1463 proteins, Olink platform).^17^ To ensure the robustness of the instruments (pQTLs), we attempted to replicate the MR results using 3 independent pQTL data sets: Atherosclerosis Risk in the Community (European and African ancestries; 9084 participants, 4657 proteins, SOMAScan platform),^18^ deCODE (35 559 participants, 4907 proteins, SOMAScan platform),^19^ and INTERVAL (3301 participants, 3622 proteins, SOMAScan platform).^20^

MR for subsequent stroke MACE and AIS was performed on the multiancestry meta-analysis. MR for incident stroke AIS was also performed on the largest known stroke GWAS published.^4^ MR analyses were performed using the TwoSampleMR R package.^21^ The pQTL data sets were meta-analyzed across ancestries.^17,19,20^ We used a 2-sample MR framework to estimate the putative causal effect of genetically proxied protein abundance to incident and subsequent stroke. MR estimates were generated using the Wald ratio method for instruments consisting of single-nucleotide polymorphisms (SNPs), which included all of our instruments. MR relies on 3 assumptions for identifying a putative causal effect, which were tested against (Supplemental Material).

#### Colocalization

Colocalization is a phenomenon whereby genetic factors at a particular locus are shared between ≥2 traits. The package coloc was used to assess whether 2 association signals are consistent with a shared causal variant.^7^ We assessed the posterior probabilities of whether the analyzed SNPs share the same causal variant (known as H4),^7^ where H4≥80% indicates strong evidence and 80%>H4≥60% indicates moderate evidence of colocalization.

#### Collider Bias Analysis and Correction

We tested whether SNPs used in the MR were associated with the published stroke incidence to determine the potential for collider bias and ran MR against both the uncorrected meta-analyzed GWAS and the Slope-Hunter adjusted meta-analyzed GWAS, as explained previously.

### Comparison of Results Against Known Druggable Targets

Once significant SNPs from GWAS results and proteins from MR results were identified, we cross-referenced these with known existing SNPs, as well as existing literature around stroke onset and progression. Finally, we compared the results from the pQTL MR against known druggable targets from Open Targets.^22^

### Reporting

This article follows the STROBE-MR (Strengthening the Reporting of Observational Studies in Epidemiology) reporting guideline.^23^ This study was approved by both UKB (Research Ethics Committee reference: 21/NW/0157; Integrated Research Application System project ID: 299116; project number: 81499) and MVP (Veterans Affairs Central institutional review board number: 16-06; MVP grant reference: I01BX004821).

## RESULTS

### Genome-Wide Association Studies

After exclusions based on ancestry and relatedness, 93 422 individuals who had an incident stroke across the UKB and MVP were analyzed (86 237 for MVP and 7185 for UKB), among whom 51 929 had subsequent MACE (50 631 for MVP and 1298 for UKB) and 45 120 had subsequent AIS (44 854 for MVP and 266 for UKB). Stroke cases were older, more commonly male, with a higher proportion of smokers and individuals with hypertension, type 2 diabetes, antihypertensive use, and lipid-lowering medication use than individuals who had never experienced an AIS (Table S1).

**Figure 2.**
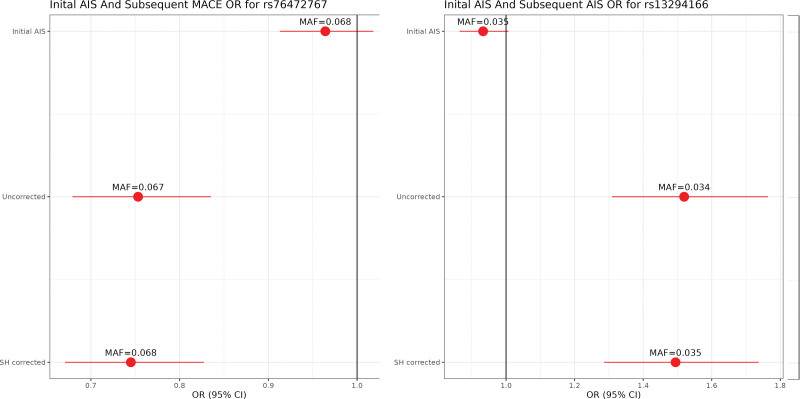
**Forest plots of initial acute ischemic stroke (AIS) and corrected and uncorrected subsequent events for the 2 genome-wide significant single-nucleotide polymorphisms.** Generalized linear mixed-effects regression models were used to test for associations between minor alleles and subsequent major acute cardiovascular events (MACE) in patients with stroke, adjusted for the first 10 genetic principal components. MAF indicates minor allele frequency; OR, odds ratio; and SH corrected, result corrected by Slope-Hunter.

There were no genome-wide significant associations in the multiancestry meta-analysis for subsequent AIS or MACE events (Figures S1 and S2), but we did observe 2 genome-wide significant (*P*<5×10^−8^) genetic variants in specific ancestry analyses: rs76472767 near gene *RNF220* on chromosome 1 in the African ancestry GWAS for subsequent MACE (Slope-Hunter corrected *P*=3.69×10^−8^) and rs13294166 near gene *LINC01492* on chromosome 9 in the African ancestry GWAS for subsequent AIS (uncorrected *P*=3.77×10^−8^; Figure 2; Table S2; Figure S3). Neither of these 2 SNPs are eQTLs (expression quantitative trait loci). For these 2 associations, we compared the results before and after Slope-Hunter correction, as well as with the results in the incident AIS. We observed that none of the significantly associated variants were associated with incident AIS, and, therefore, the Slope-Hunter correction for collider bias may not have been necessary, and the uncorrected results may be considered unbiased (Figure 2). However, the Slope-Hunter correction for collider bias may lead to slight differences in the results.

We tested for tissue enrichment of subsequent MACE GWAS signals using expression data with MAGMA (Multi-Marker Analysis of GenoMic Annotation) in Functional Mapping and Annotation of GWAS. However, no tissues were expressed above any statistically significant threshold (Figure S4).

### Expected Versus Observed Replication

We sought to determine whether the genetic factors for incident stroke were also relevant for subsequent stroke. Of the 91 SNPs previously reported to associate with incident stroke, we observed that 77 replicated in our incidence GWAS at a nominal *P*<0.05 (91 expected given the power difference of 4×10^−29^). By contrast, our subsequent MACE GWAS replicated only 33 associations at a nominal *P*<0.05 (compared with 82 expected, power difference, 3×10^−35^), suggesting there is overlapping, but also distinct genetic etiology of incident stroke and subsequent MACE. This pattern was consistent when using collider bias corrected results (Table S3).

### MR Against pQTL Data

Subsequent MACE results include a high proportion of subsequent AIS; therefore, the MR effect was generally of similar magnitude and direction but due to lower sample size, had larger *P* values and wider CIs; therefore, we focus our MR study on the subsequent MACE results (full results in Table S4). We observed 6 genes for incident stroke and 2 genes for subsequent MACE that have a significant MR result (adjusted *P*<0.05) and supporting colocalization evidence (posterior probability H4, >60%; Table). For all 6 genes, all MR results are based on single instrumental variant since only 1 cis-pQTL was available and the Wald ratio was used.

**Table.**
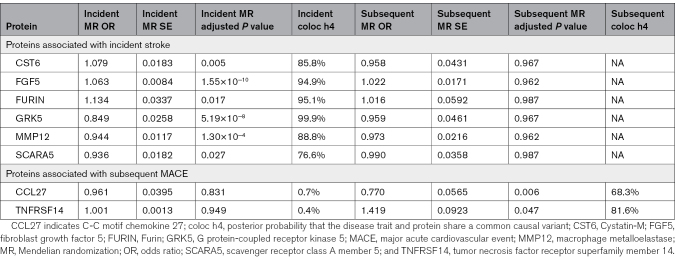
MR and Colocalization Results From the UK Biobank Pharma Proteomics Project Protein Quantitative Trait Loci Data Set Against Both Incident and Subsequent MACEs

#### Incident pQTL Results

We identified 6 proteins (CST6, FGF5, FURIN, GRK5, MMP12, and SCARA5) with evidence for a putative causal effect on incident AIS (adjusted *P*<0.05). However, none of these showed evidence for a putative causal effect on subsequent MACE (Table S4). All except SCARA5 showed strong evidence for colocalization, while SCARA5 showed moderate evidence of colocalization (Figure S5).

#### Subsequent MACE pQTL Results

Two proteins (CCL27 and TNFRSF14) showed evidence for a putative causal effect on subsequent MACE (adjusted *P*<0.05; Table). Neither of these proteins showed a putative causal effect on incident stroke. Genetically predicted higher levels of CCL27 showed evidence of a protective effect against subsequent MACE (odds ratio, 0.77 [95% CI, 0.66–0.88]; *P*=0.00618). In contrast, higher predicted TNFRSF14 levels increased the risk of subsequent MACE (odds ratio, 1.419 [95% CI, 1.24–1.60]; *P*=0.0469). After collider bias correction using Slope-Hunter, the MR results for CCL27 and TNFRSF14 were not significantly affected (CCL27: 0.77 [95% CI, 0.65–0.89], *P*=0.00588; TNFRSF14: 1.419 [95% CI, 1.24–1.60], *P*=0.0869; Table S4). However, as these specific variants were not associated with incident disease (Table), the potential for collider bias was minimal. Both proteins implicated have a role in inflammation. TNFRSF14 has been associated with proinflammatory factors in tumors and has poor prognosis for follicular lymphoma,^24^ while CCL27 has been associated with inflammatory skin diseases like atopic dermatitis, contact dermatitis, and psoriasis, and increased CCL27 secretion is shown to cause inflammation.^25^

There was moderate evidence for colocalization of CCL27 (H4, 68%) and strong evidence for TNFRSF14 (H4, 81%; Table; Table S5; Figures S6 and S7). The assignment of the remaining probability mostly to H1 (representing association only with protein trait and not subsequent MACE) suggests this analysis has limited power, rather than suggesting there are independent effects that do not colocalize (H3, the posterior probability that the disease trait and protein have 2 independent SNPs).

#### Verification of pQTL Instruments Using Other Data Sets

To verify that the protein instruments identified in UKB Pharma Proteomics Project were valid, we replicated the MR results using 3 other independent pQTL data sets (Atherosclerosis Risk in the Community, deCODE, and INTERVAL). For 5 of the 9 significant MR results, MR using independent pQTL data sets showed consistent putative causal effects (Table S6). Due to the differing power of the pQTL data sets, pQTLs were filtered for having an F statistic value above 10 (Table S7).

#### Multiancestry Comparison of MR Results and Meta-Analysis

**Figure 3.**
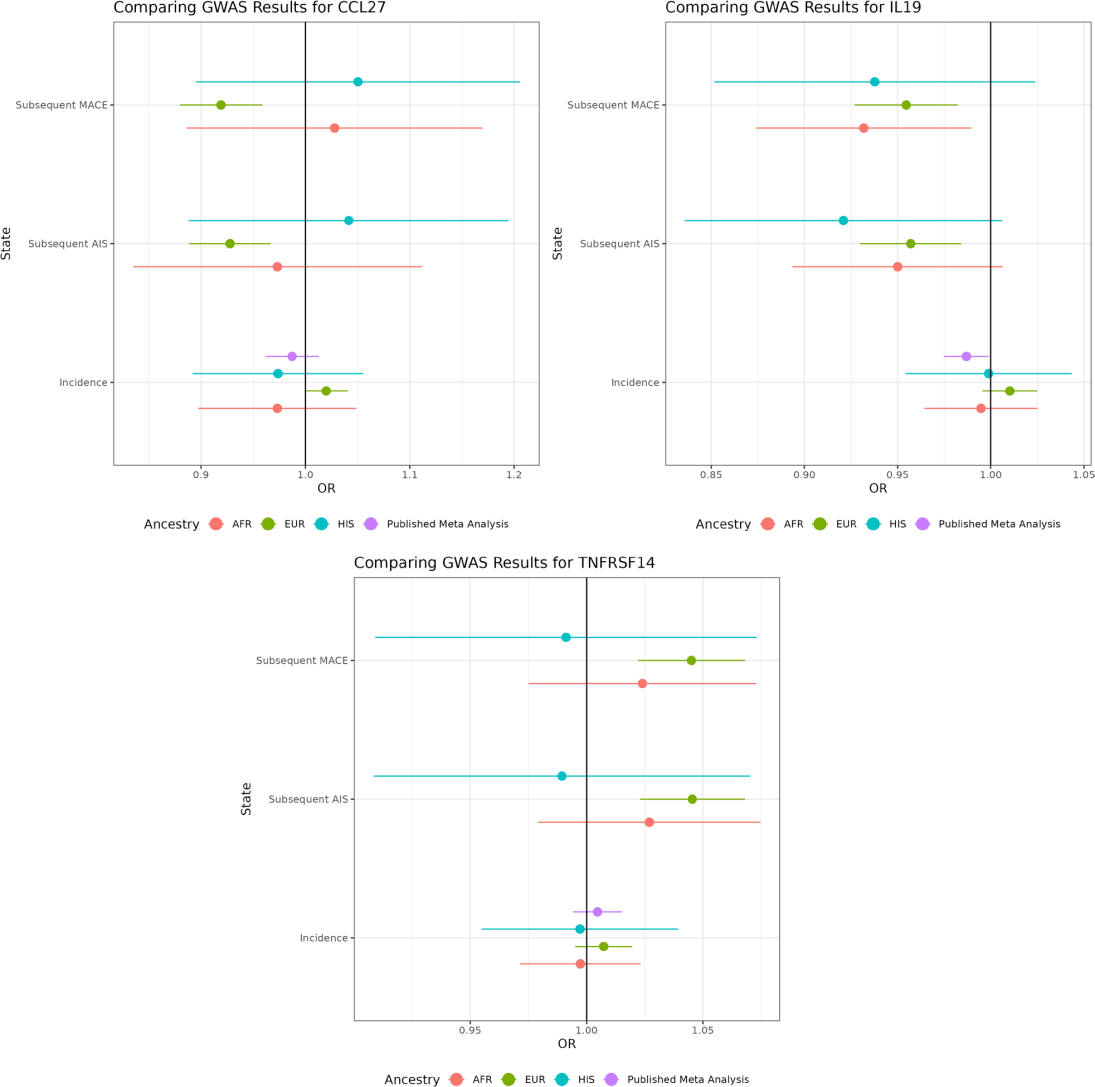
**Significant Mendelian randomization results in subsequent stroke states by ancestry, as well as a comparison to incident stroke (Cochran Q p≥0.1).** AFR indicates African ancestry; AIS, acute ischemic stroke; CCL27, C-C motif chemokine 27; EUR, European; GWAS, genome-wide association study; HIS, Hispanic; IL-19, interleukin-19; MACE, major acute cardiovascular event; OR, odds ratio; and TNFRSF14, tumor necrosis factor receptor superfamily member 14.

There is little evidence that the 2 proteins (CCL27 and TNFRSF14) reported as having putative causal effects on subsequent MACE have different effects across the 3 ancestries tested. However, this is primarily due to the wide CIs (and small sample sizes) within Hispanic and African subgroups (Figure 3).

#### Comparing MR Results Against Potential Druggable Targets

Of the 720 genes related to ischemic stroke in Open Targets,^22^ 103 had instrumentable protein products (*P*<1×10^−11^) in UKB Pharma Proteomics Project.^17^ Five of these met a false discovery rate–adjusted significance threshold in MR for incident stroke (ANGPT1, FGF5, FURIN, MMP12, and TFPI) but none in the MR of subsequent MACE. Of the 5 targets with evidence of a causal effect on incidence, 4 were previously identified as putatively causal for incident stroke in MR studies,^24,25^ while FURIN is novel. ANGPT1, TFPI, and MMP12 have evidence of a causal effect on incident AIS, while FURIN and FGF5 have existing genetic associations. Using the Therapeutic Target Database^26^ to find existing therapeutic drugs for these genes, we determined that TFPI and ANGPT1 have phase 3 clinical trials associated with them, MMP12 has a phase 1 trial, and FURIN has a preclinical trial. None of the drugs that started clinical trials were designed for stroke (Table S8). For markers associated with subsequent MACE, currently, there are no clinical trials of drugs targeting TNFRSF14 and CCL27.

## DISCUSSION

We observed 2 proteins (CCL27 and TNFRSF14) associated with subsequent stroke events that have a role in inflammation. There exists a link between inflammation and stroke. While the immune response starts locally, inflammatory mediators propagate, which leads to a systemic inflammatory response, followed by immunosuppression.^27^ Changes in TNF (tumor necrosis factor) and IL (interleukin)-6 levels have been observed in patients at the onset of stroke.^28^ This response may be due to a state of immunodepression that occurs poststroke, as there are increased risks of poststroke infections.^27^ There is increasing evidence that greater inflammation is associated with AIS progression. It is unclear whether inflammation is transitory, related to the severity of the ischemia, and the ischemia-inflammation association poststroke is not well characterized.^29^

The discovery of 2 proteins having a predicted causal effect on subsequent MACE after stroke suggests that inflammation is a contributing factor to subsequent MACE outcomes after incident stroke AIS.^30,31^ TNFRSF14 (also known as HVEM) signals via the 9/3 pathway, which has a role in immune cell survival. TNFRSF14 is a receptor for 4 ligands: TNFSF14 (LIGHT), LTA, BTLA, and CD160. First 2 are TNF cytokines, and second 2 are immunoglobulin-related membrane proteins. TNFRSF14 has been shown to contribute to plaque destabilization and rupture.^32^ The LIGHT protein is known to have prognostic predictive value for composite cardiovascular events.^33^ The TNF-α family also has been suggested as being a risk factor to stroke.^34^ CD160 has been shown to be a potential indicator of the progression of atherosclerosis.^35^ Plasma measures of 4 of these 5 ligands were available in the UKB proteomics data, but (despite having strong instruments available, F>500) none had a causal effect on subsequent MACE (LTA, *P*=0.987; CD160, *P*=0.990; TNFSF14, *P*=0.941; TRAF2, *P*=0.99; full results in Table S4). CCL27 is a cytokine involved in maintaining immune homeostasis in barrier tissues, and a lack of CCL27 in mice has shown an increased infiltration of CCR10^+^ T cells in reproductive tracts that displayed signs of inflammation.^36^

A third protein that did not reach the statistical significance threshold for reporting, IL-19, showed slighty weaker evidence of a causal effect on subsequent MACE (odds ratio, 0.878 [95% CI, 0.81–0.94]; adjusted *P*=0.053; coloc h4, 69%) and also no effect in incident AIS (odds ratio, 0.963 [95% CI, 0.92–1.00]; adjusted *P*=0.496). IL-19 is an anti-inflammatory marker^37^ and diminishes cerebral infarction and neurological deficits following cerebral ischemia in mice, potentially through the elevated expression of genes related to proinflammatory cytokines.^38^ As increased IL-19 levels appear to be an anti-inflammatory marker, and increased TNFRSF14 levels show a role in immune cell survival,^39^ this leads to the notion of inflammation as a contributor to subsequent MACE outcomes. As CCL27 is used in maintaining immune homeostasis in barrier tissues, it is more difficult to ascertain what a negative effect size could infer without further investigation.

We observed genetic variants that appear exclusively associated with subsequent MACE and AIS after an incident AIS. This might imply novel biological insights into the disease progression of stroke. We observed that all 6 proteins that show a putatively causal effect on incident AIS do not appear to affect subsequent strokes. All individuals in this study were diagnosed, treated, and likely given blood pressure medications, statins, or both, which could mask an effect on subsequent stroke risk.

Of the 5 targets identified by MR from the drug target list in Open Targets (ANGPT1, FGF5, FURIN, MMP12, and TFPI), none are associated with subsequent MACE. This suggests that these proteins may be important therapeutic targets to reduce risk for incident AIS but not for subsequent MACE. Existing targets in Open Targets is, in part, populated by correlations in genetic association, thus why they initially became candidate targets. We postulate that genetic variants and genes for incident stroke are not good targets for drug discovery of subsequent stroke events.

We note that while the Cochran Q statistic for heterogeneity did not show evidence for different effects between ancestries (Table S9), the difference in sample sizes between ancestries remains large. Results from European ancestry were overall much stronger due to higher power. More data around individuals of non-European ancestries are necessary to investigate this further.

We had several limitations to our study. First, MACE is defined as a combination of myocardial infarction, AIS/transient ischemic attack, and ASCVD death. For incident MACE in MVP, we have previously observed that myocardial infarction accounts for a larger proportion of the MACE phenotype compared with AIS/transient ischemic attack.^40^ However, our subsequent cohort has a smaller proportion of myocardial infarction events than expected in the MACE phenotype, most likely due to selection of subsequent MACE by incident AIS status, which might limit the scope of these results to the general population (Table S10). This is likely due to the selection on incident AIS. Second, despite analyzing relatively large data sets for disease progression, our results have limited statistical power due to sample sizes. Third, as cis-only pQTL data sets are normally instrumented by a single SNP, the Wald ratio is the only available means of estimation for MR, which restricts the type of sensitivity analyses we can perform. Colocalization reduces the risk of confounding by linkage disequilibrium as it requires the presence of a common causal variant responsible for both traits but cannot exclude potential horizontal pleiotropy, and other sources of pleiotropy could not be tested in this study. The MVP cohort also has a higher proportion of men compared with women; hence the results may not be generalizable to both sexes. Finally, a lack of sufficient sample sizes and access to data in ancestries other than European, African, or Hispanic means that we cannot ascertain whether these results are generalizable across all ancestries or whether there are genetic differences by ancestry. All individuals who were diagnosed with stroke will have likely been put on common preventative medication for subsequent stroke; this treatment may be altering the GWAS of progression results; however, no data are available to compare against individuals who have been diagnosed with stroke but have not been treated.

## CONCLUSIONS

We observed 2 novel SNPs associated with subsequent stroke events in African ancestry individuals that warrant further replication. We also performed MR to identify putative causal proteins for risk of subsequent MACE in patients with stroke. We observed putatively causal evidence for 2 novel proteins (CCL27 and TNFRS14) associated with subsequent MACE risk in pQTL, suggesting that inflammation is a contributing factor to subsequent MACE outcomes after incident stroke AIS.

## ARTICLE INFORMATION

### Acknowledgments

The authors acknowledge the VA Million Veteran Program (MVP) participants. The UK Biobank (UKB) data were obtained under the UKB resource application 81499. The authors thank the participants of MVP and UKB studies. Drs Paternoster, Cho, and Peloso jointly supervised the research.

### Sources of Funding

This study was supported by the National Institute for Health and Care Research Bristol Biomedical Research Centre. This research is based on data from the Million Veteran Program (MVP), Office of Research and Development, Veterans Health Administration, and was supported by Veterans Affairs Merit Awards BX004821 and CX001025. This publication does not represent the views of the Department of Veteran Affairs or the US Government. Please see the Supplemental Material for MVP core acknowledgements. This publication is the work of the authors who will serve as guarantors for the contents of this article. The views expressed in this publication are those of the authors and not necessarily those of the NHS, the National Institute for Health Research. A. Elmore, Dr Paternoster, Dr Gaunt, Dr Davey Smith, Dr Hemani, and Dr Hartley receive support from the UK Medical Research Council Integrative Epidemiology Unit at the University of Bristol (MC_UU_00011/4, MC_UU_00011/1, MC_UU_00032/01, MC_UU_00032/03). Dr Aparicio is supported by the American Academy of Neurology Career Development Award and the National Institutes of Health R01NS017950.

### Disclosures

Dr Tilling reports grants from the National Institute for Health and Care Research; grants from Wellcome Trust; and grants from Medical Research Council. Dr Gaunt reports grants from the National Institute for Health and Care Research (UK); grants from Biogen; grants from UK Medical Research Council; and grants from GlaxoSmithKline. Dr Gaziano reports grants from the US Department of Veterans Affairs. Dr Davey Smith reports grants from the Medical Research Council. Dr Peloso reports support from Veterans Health Administration; employment by Boston University; compensation from the American Heart Association for consultant services; and grants from the National Institutes of Health. Dr Hartley started working for Novo Nordisk after contributing to this article. The other authors report no conflicts.

### Supplemental Material

Supplemental Methods

Figures S1–S7

Tables S1–S10

STROKE-MR Checklist

VA Million Veteran Program: Core Acknowledgement

References 41–47

## Supplementary Material


