# Supplementary material for: Protein Identification for Stroke Progression via Mendelian Randomization in Million Veteran Program and UK Biobank
Source: Stroke. 2024 Jul 23;55(8):2045–54. doi: 10.1161/STROKEAHA.124.047103 (PMC11259242; doi:10.1161/STROKEAHA.124.047103)
Supplement: Supplementary file 2 [file str-55-2045-s002.pdf]

Supplemental Material

Supplementary Methods

International Classification of Disease (ICD) codes for AIS/MACE phenotype in MVP and UKB

The following ICD codes were used to define AIS in MVP:

High probability ICD-9 and -10 coding for stroke and TIA in MVP

Cerebrovascular disease condition	ICD-9	Source(s)	ICD-10	Source(s)
Acute ischemic stroke	433.x1, 434.x, 436.x, excluding 434.x0	Tirschwell David L, et al. *Stroke*. 2002;33(10):2465-2470. PMID: 12364739.* "Tirschwell criteria"	I63.x (cerebral infarction)	PMID: 16020772**, †
Intracerebral Hemorrhage	431.x		I61.x	PMID: 33061648†† PMID: 23735951€ PMID: 31955830€€ Reviews: PMID: 22262598 and 26292280@
Subarachnoid Hemorrhage	430.x		I60.x	
TIA	435.x		G45.x, exc G45.4	PMID: 12063102@@
**Stroke to the retina (a type of acute ischemic stroke, using newer definitions)**				
Retinal vascular occlusion/Central retina artery occlusion	362.3		H34.1	
**Coding that is potentially insensitive for true acute stroke**				
Other CVA (undefined ischemic or hemorrhagic)	437.x – "Other and ill-defined cerebrovascular disease"		I64.x (stroke, not specified as hemorrhage or infarction)	PMID: 33061648: I64 does not exist in ICD-10-CM. Including I64 might not lower the PPV for AIS substantially@#
			I67.x (Other cerebrovascular disease)	Insensitive for AIS
Traumatic brain injury – consider excluding case if TBI is primary ICD code	800-804, 850-854			
Rehabilitation care – consider excluding case if rehab care is primary ICD code	V57			

* N=206. ICD-9 PPV: AIS 90%, ICH 80%, SAH 86%; overall (AIS/ICH/SAH) kappa 0.79 (highly substantial agreement)

** Excluded: "433.x0 (occlusion and stenosis of precerebral arteries without mention of cerebral infarction), 434.x0 (occlusion of cerebral arteries without mention of cerebral infarction), 437.x (other and ill-defined cerebrovascular disease), 438.x (late effects of cerebrovascular disease), I65.x (occlusion and stenosis of precerebral arteries not resulting in cerebral infarction), I66.x (occlusion and stenosis of cerebral arteries not resulting in cerebral infarction), I67.x (other cerebrovascular diseases), I69.x (sequalae of cerebrovascular disease), and G45.4 (transient global amnesia)." Can remove ICD-9 436 in acute ischemic stroke, to be very conservative (PMID: 26292280). Or include both ICD-9 436 and ICD-10 I64 to be less conservative (PMID: 26292280).

† N=256. ICD-10 overall (AIS/ICH/SAH/TIA) kappa 0.89 (excellent)
†† N=1,235. ICD-10 I63* PPV for AIS: 92.7% (Taiwan)
€ N=465, ICD-10 I63* PPV for AIS: 85% (France)
€€ N=1,551. I639 (cerebral infarction, unspecified) had highest false positive rate (67.6%)
@Systematic review. ICD-10 I63* PPV for AIS: ≥82% in most studies; ICD-10 I63*+I64* for AIS: ≥75%; **Fatal stroke PPV ≥87% in most studies.**
@@ TIA codes should be used with caution, typically avoid for acute stroke events. E.g. N=134. ICD-10 G45 PPV, only 60.4% verified on medical record review (Denmark)
# More broadly, using general coding ICD-9 430–438 and ICD-10 I60-69 for acute stroke should be discouraged (PPV ≤ 68% in most studies).

Phenotype Definitions

Incident stroke was defined as any diagnosis of AIS or transient ischaemic attack (TIA) using hospital linked data. People who experienced their initial stroke more than one year prior to recruitment were excluded from our stroke phenotype. Specific International Classification of Disease (ICD) codes used for both MVP and UKB can be found in the supplementary information.

Subsequent AIS/TIA was defined as any secondary diagnosis of AIS/TIA at least 90 days after the incident diagnosis, to avoid recoding of the primary event, and would be considered events after the acute phase of an incident AIS/TIA. Individuals who did not survive at least thirty days after their incident stroke diagnosis were excluded from analyses of subsequent outcomes, to emulate a target clinical trial. Subsequent MACE was defined as any subsequent stroke, myocardial infarction (MI), or death due to atherosclerotic cardiovascular disease (ASCVD), with the first event that happens after 90 days used to construct the MACE phenotype. Vascular disease events occurring before or after the initial stroke were excluded, but events greater than 90 days post stroke were included.

MI in MVP was identified using ICD10 codes I2[1-2]*, and I25.2* and ICD9 codes 410* and 411.0*. ASCVD death in MVP was identified using the ICD10 codes I10*, I11*, I13*, I16*, I2[0-5]*, I46*, I63*, I66[0-9]9, I67*, I70* or I7[4-5]* and ICD9 codes 41[0-2]*, 414*, or G45* as the cause of death. Date of subsequent MACE was taken as the corresponding date of death starting from a year before enrollment or the date of MI starting from a year before enrollment.

AIS in UK Biobank was defined as any ICD10 code of I63* or any ICD9 code 433*, 434* or 4371. TIA was defined as any ICD10 code of G45.8 or G45.9 or an ICD9 code 4359. The corresponding date of diagnosis was extracted from the corresponding 41280/41281 fields.
MI was identified using ICD10 codes I21*, I22* or I25.2 and ICD9 codes 4109, 4129 or 4119. ASCVD death was defined as ICD10 codes I10, I11*, I13*, I20*, I21*, I22*, I23*, I24*, I25*, I46*, I63*, I66*, I67*, I70*, I74* or G45*, and ICD9 codes 414*, 4109, 4129 or 4119 as the cause of death using the death registry data (fields 40001 and 40002). The date of subsequent MACE outcomes was defined as either the date in the corresponding 41280/1 columns for HES data or the date of death for ASCVD death.

The discrepancies in ICD codes between MVP and UK Biobank are due to differences in data labelling and data availability between the two datasets.

UK Biobank (UKB)

UKB is a prospective cohort study with over 500,000 participants aged 40-69 (average 56.5) years when recruited in 2006-2010 and 54% of participants are women[8]. Genotype imputation to a reference set combining the UK10K haplotype and HRC reference panels[41,42] was performed using IMPUTE2 algorithms[43]. The analyses presented here were restricted to autosomal variants with a MAF > 0.01 and Imputation Info > 0.7, with MAF and Info scores having been recalculated on an in-house derived 'European' subset. Prior to phasing, multiallelic SNPs or those with MAF ≤1% were removed. Phasing of genotype data was performed using a modified version of the SHAPEIT2 algorithm[44]. In order to mitigate potential confounding arising from population stratification, participants were excluded from the analysis for the subsequent reasons: withdrawal of consent, genetic-reported sex mismatch, sex chromosome aneuploidy, genotyping of poor quality (missing rate exceeding 1.5%), or significant relatedness (in excess of >10 putative third-degree relatives).

GWAS were performed using linear mixed models using BOLT-LMM[44], with adjustment for age at first stroke, sex, genotyping chip and 10 ancestry principal components (PCs). This was performed on individuals of European genetic ancestry, including minimally related individuals. SNP-based heritability was estimated from the summary statistics using LD score regression, evidence for potential residual confounding was assessed using the LD score regression intercept and Q-Q plots. Results from BOLT-LMM were adjusted from quantitative scale to traditional odds ratios, so it could be compared with results from the MVP data, as suggested by the BOLT manual[44].

Million Veteran Program (MVP)

MVP is a continually growing cohort of over 850,000 participants by 2021[9], 8% women, with an average age of 61.9 years[10]. Consented individuals in the MVP were genotyped using a customized Affymetrix Axiom Biobank Array. Imputation was performed using a 1000 Genomes Project Phase 3 v.5 reference panel after pre-phasing with EAGLE[42], supplemented with the African Genome Resources panel[45]. Sample and variant quality control for MVP are previously described[40]. Individuals were assigned to harmonized genetic ancestry and race ethnicity (HARE) groups[46], which uses a machine learning model to predict self-identified race and ethnicity (SIRE) from principal components of genetic ancestry and was developed to facilitate ancestry-specific GWAS in the MVP. Two outcomes were also used for analyses in MVP: subsequent stroke AIS and any subsequent MACE. A Firth logistic regression was performed for the GWAS using Regenie[47], adjusting for age at first stroke, sex and the first 10 ancestry PCs. GWAS of incident stroke, subsequent stroke and MACE were performed for European, African, and Hispanic HARE groups separately. The frequency of genome wide significant SNPs were compared to the reference population in the 1000 Genomes Project[12] and the variants were excluded if the MAF in our population was different from the reference MAF by 0.2.

Collider Bias Sensitivity Analysis and Correction

Slope-Hunter assumes that SNPs can be divided into clusters based on their causal relationship with incident and subsequent events and uses SNPs associated with the incident event only to provide an estimate of the bias correction factor for the study, hence is more robust to the correlation between incident and subsequent events. However, when investigating specific SNPs and their associated regions, collider bias correction may only be necessary if there is an association of the variant with incident AIS to begin with. For that reason, we have compared Slope-Hunter adjusted results with non-Slope-Hunter adjusted results, as well as compared the results with the associated region in the incident GWAS. Each Slope-Hunter calculation was performed for each specific ancestry as the collider bias may behave differently in each subset of data.

We used the Slope-Hunter method with a default p-value threshold of 0.001 to correct the summary statistics for further analyses. We used the 1000 genomes reference panel[13] for clumping matched by ancestry group, with an r^2 threshold of 0.1.

Mendelian randomization (MR)

MR relies on three assumptions for identifying a putative causal effect[23], the genetic instrument should: 1) associate with the exposure (relevance), 2) have no shared causal factors with the outcome (independence), and 3) solely influence the outcome through the impact of the risk factor of primary concern (exclusion restriction). The relevance assumption was tested by generating the F-statistic for each instrument, where an F-statistic > 10 is evidence against weak instrument bias[24]. The exclusion restriction assumption is difficult to assess with single SNP instruments, as is common for molecular traits. Therefore, we additionally performed colocalization. Finally, to explore if there was any evidence of heterogeneity of effects between genetic ancestries, for SNPs used in MR, we compared the associations with the outcomes across ancestries.

Figure S1: Manhattan Plots of ancestry Specific and Meta Analyzed GWAS of subsequent AIS and MACE

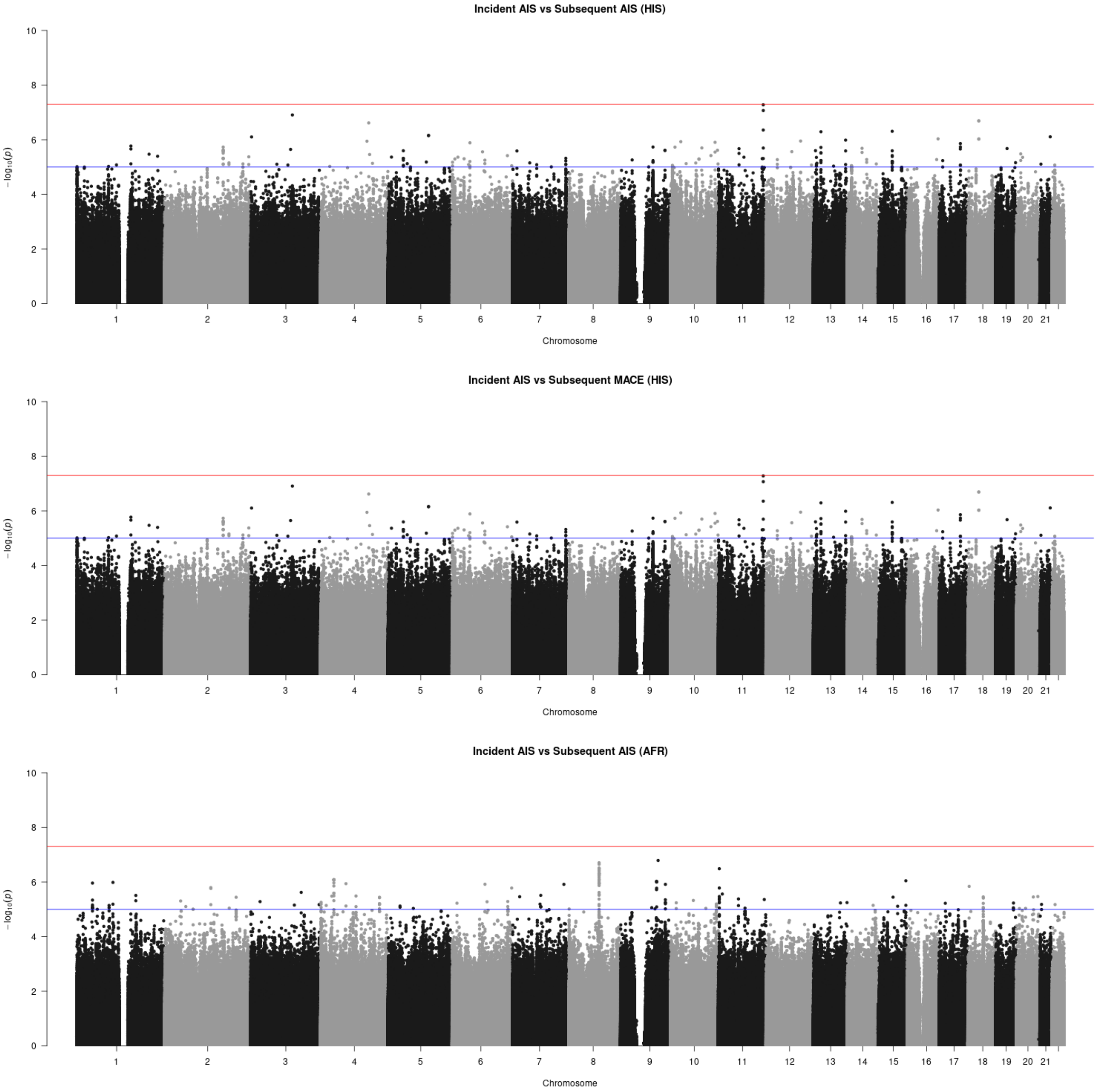

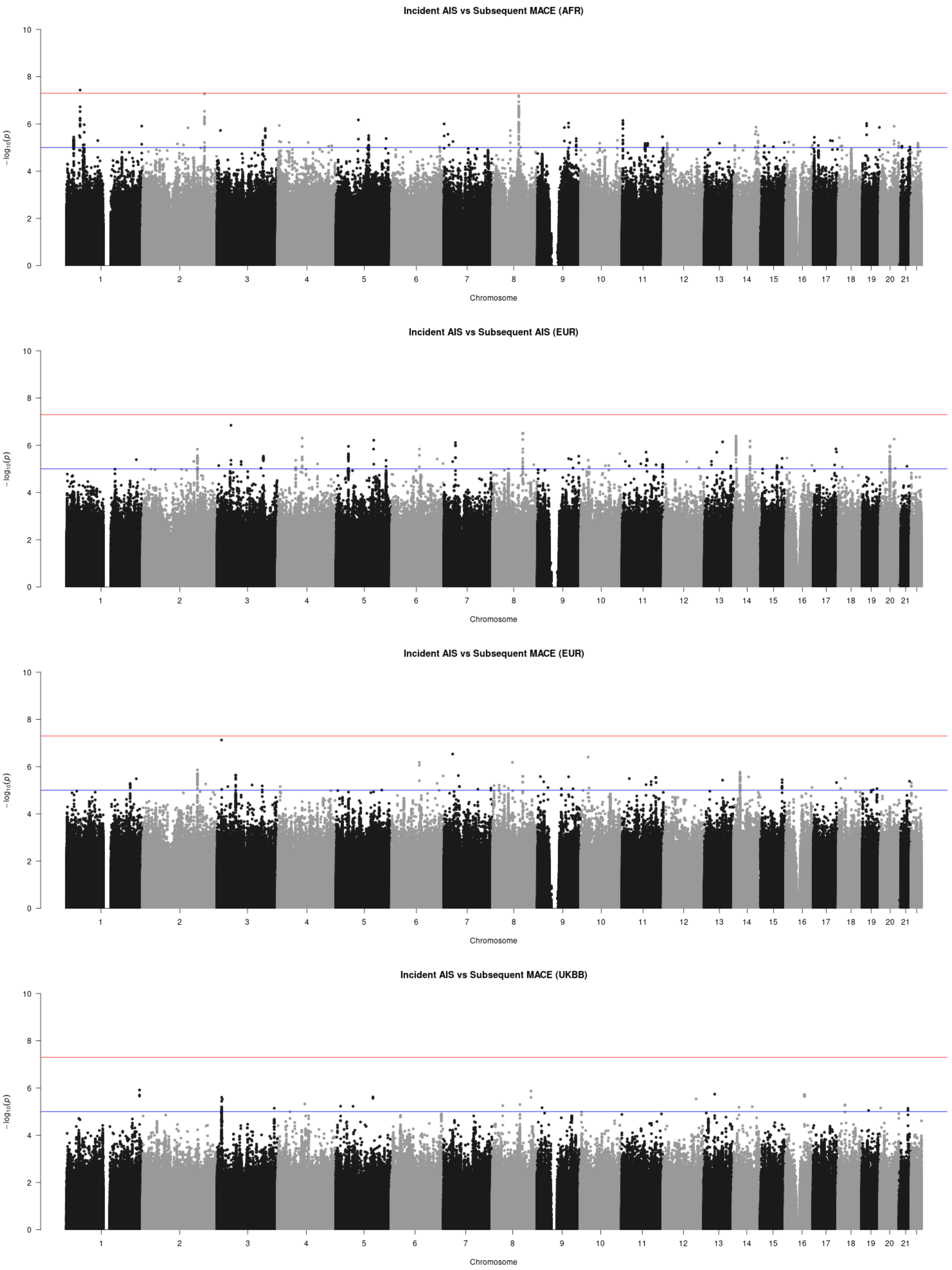
Incident AIS vs Subsequent MACE (AFR)
Incident AIS vs Subsequent AIS (EUR)
Incident AIS vs Subsequent MACE (EUR)
Incident AIS vs Subsequent MACE (UKBB)
$-\log_{10}(p)$
Chromosome

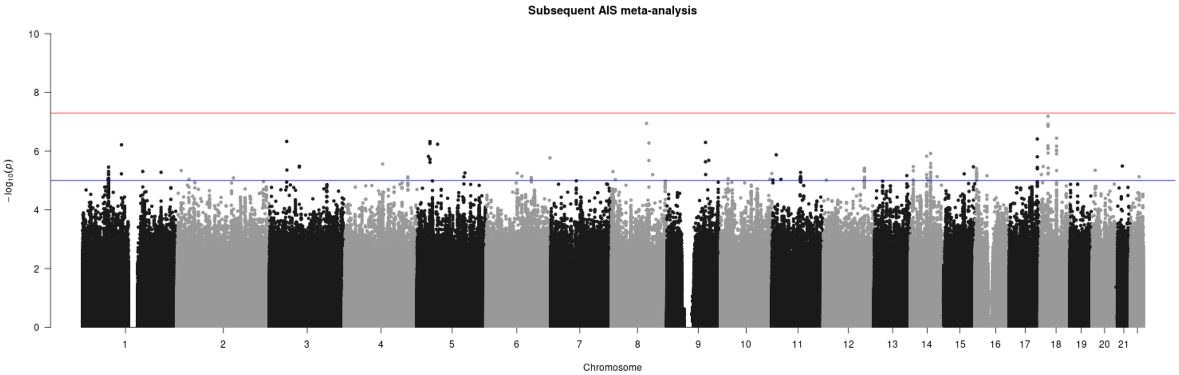
Subsequent AIS meta-analysis
10
8
6
4
2
0
$-\log_{10}(p)$
1 2 3 4 5 6 7 8 9 10 11 12 13 14 15 16 17 18 19 20 21
Chromosome

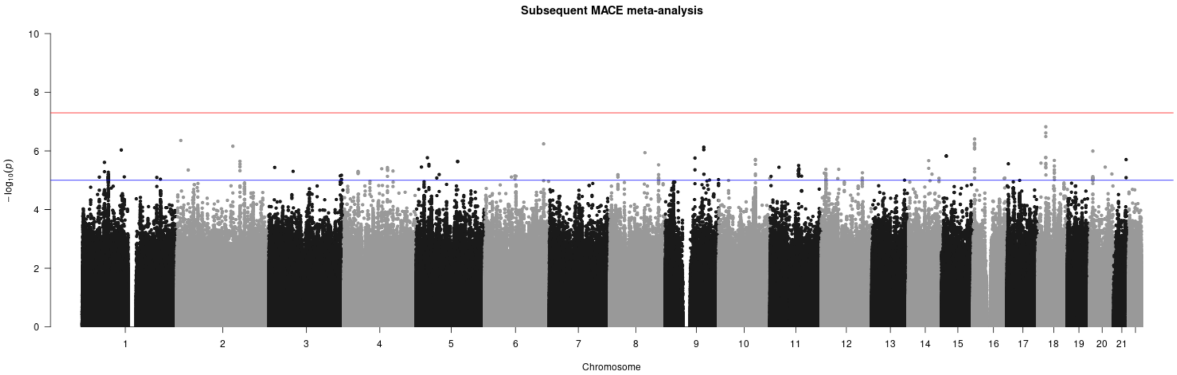
Subsequent MACE meta-analysis
10
8
6
4
2
0
$-\log_{10}(p)$
1 2 3 4 5 6 7 8 9 10 11 12 13 14 15 16 17 18 19 20 21
Chromosome

Figure S2: Genomic Lambdas and QQ plots for Slope-Hunter corrected GWAS results for all the various ancestry groups and meta-analyses for the two outcomes of subsequent AIS and subsequent MACE.

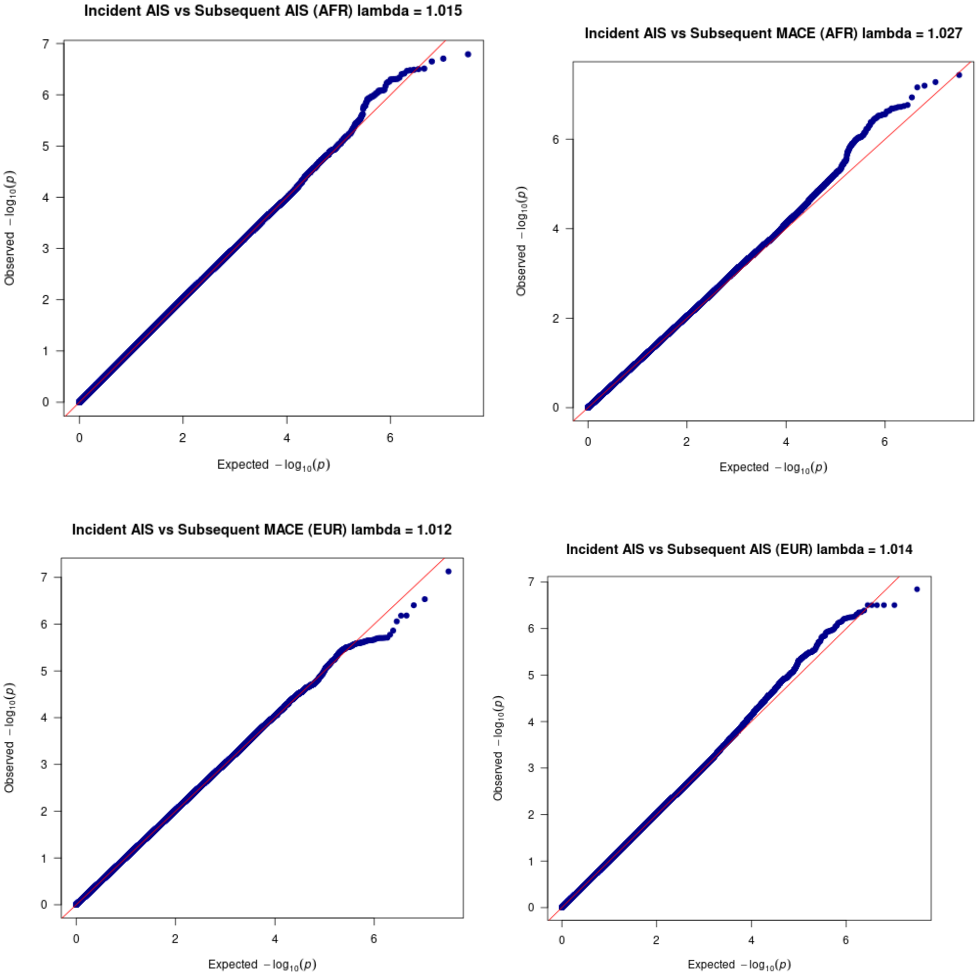

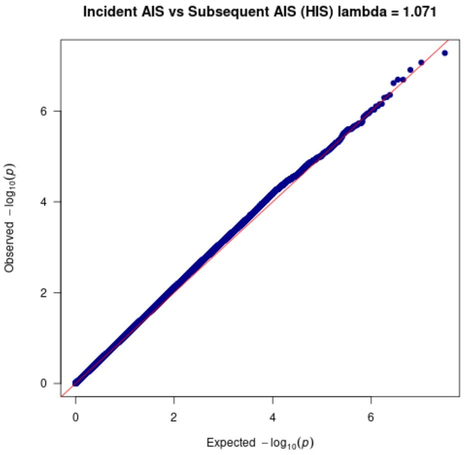
Incident AIS vs Subsequent AIS (HIS) lambda = 1.071
Observed $-\log_{10}(p)$
Expected $-\log_{10}(p)$
0
2
4
6

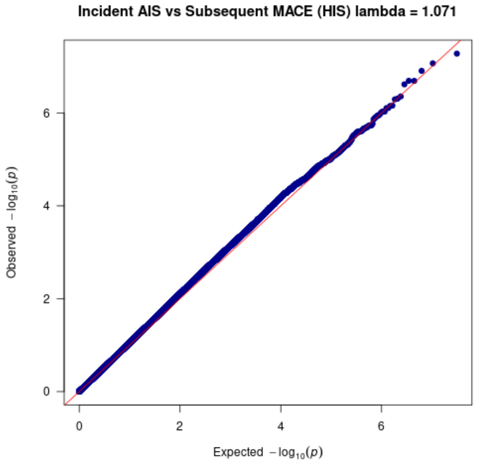
Incident AIS vs Subsequent MACE (HIS) lambda = 1.071
Observed $-\log_{10}(p)$
Expected $-\log_{10}(p)$
0
2
4
6

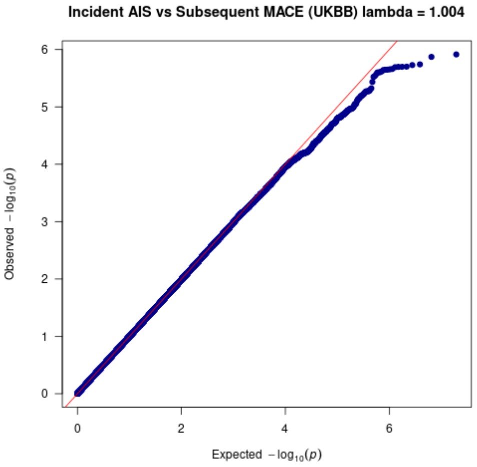
Incident AIS vs Subsequent MACE (UKBB) lambda = 1.004
Observed $-\log_{10}(p)$
Expected $-\log_{10}(p)$
0
1
2
3
4
5
6

Figure S3: Forest plots of effect sizes for all GWAS performed for the 2 SNPs associated with subsequent events.

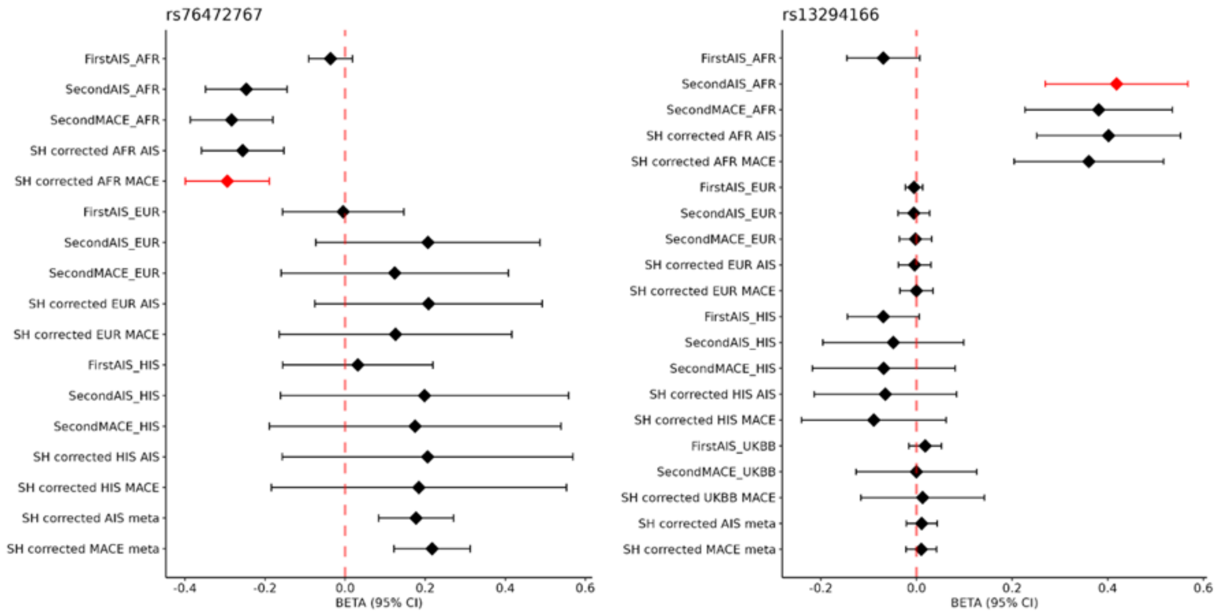

Figure S4: MAGMA Tissue Expression Analysis of Subsequent MACE GWAS

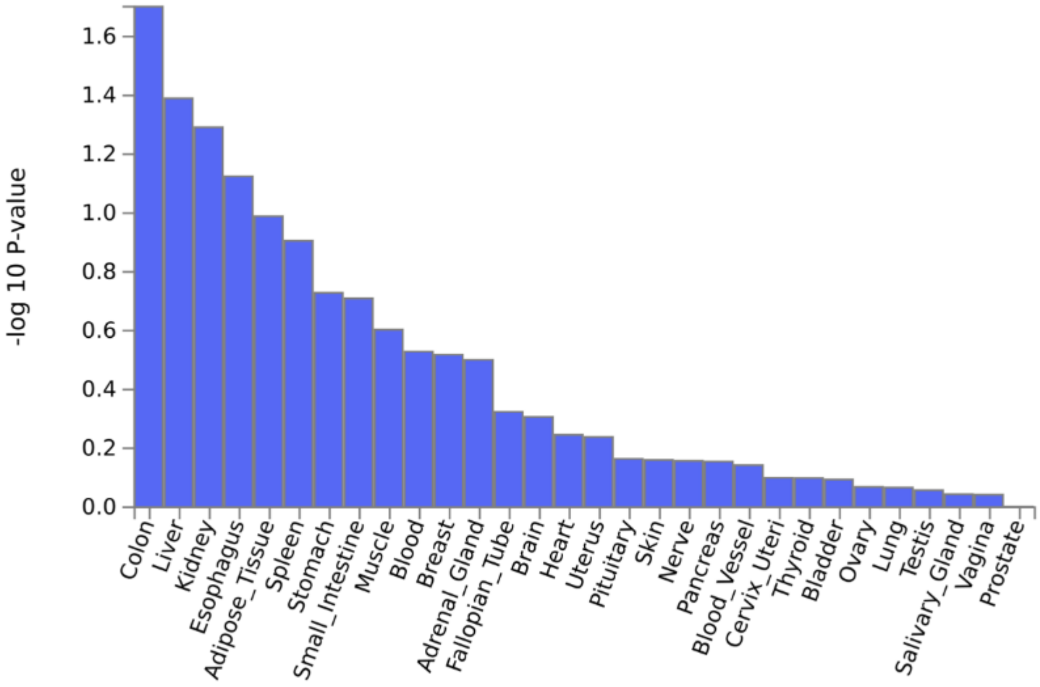

Figure S5: Miami Plots Comparing pQTL data and Incidence GWAS in areas highlighted by MR Result

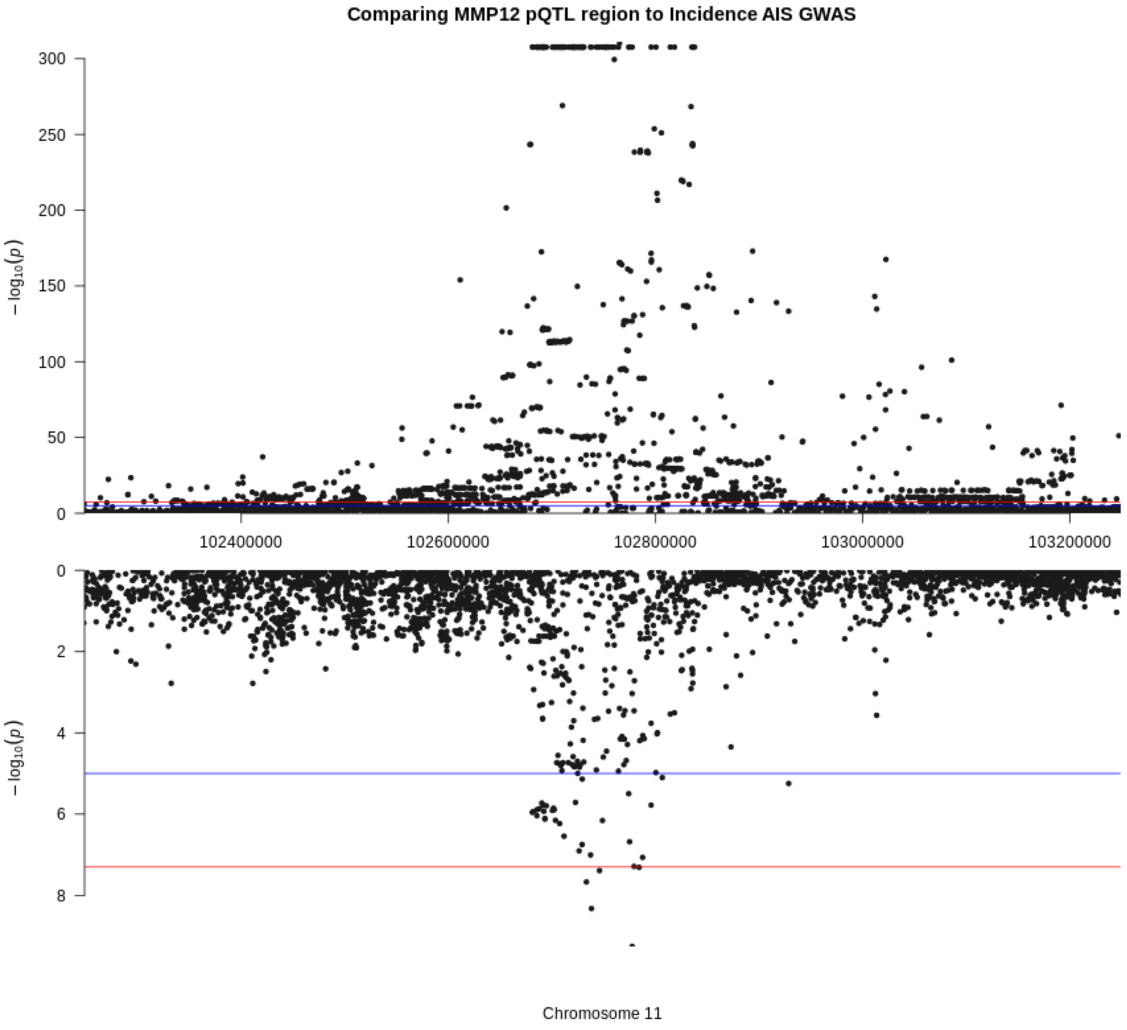

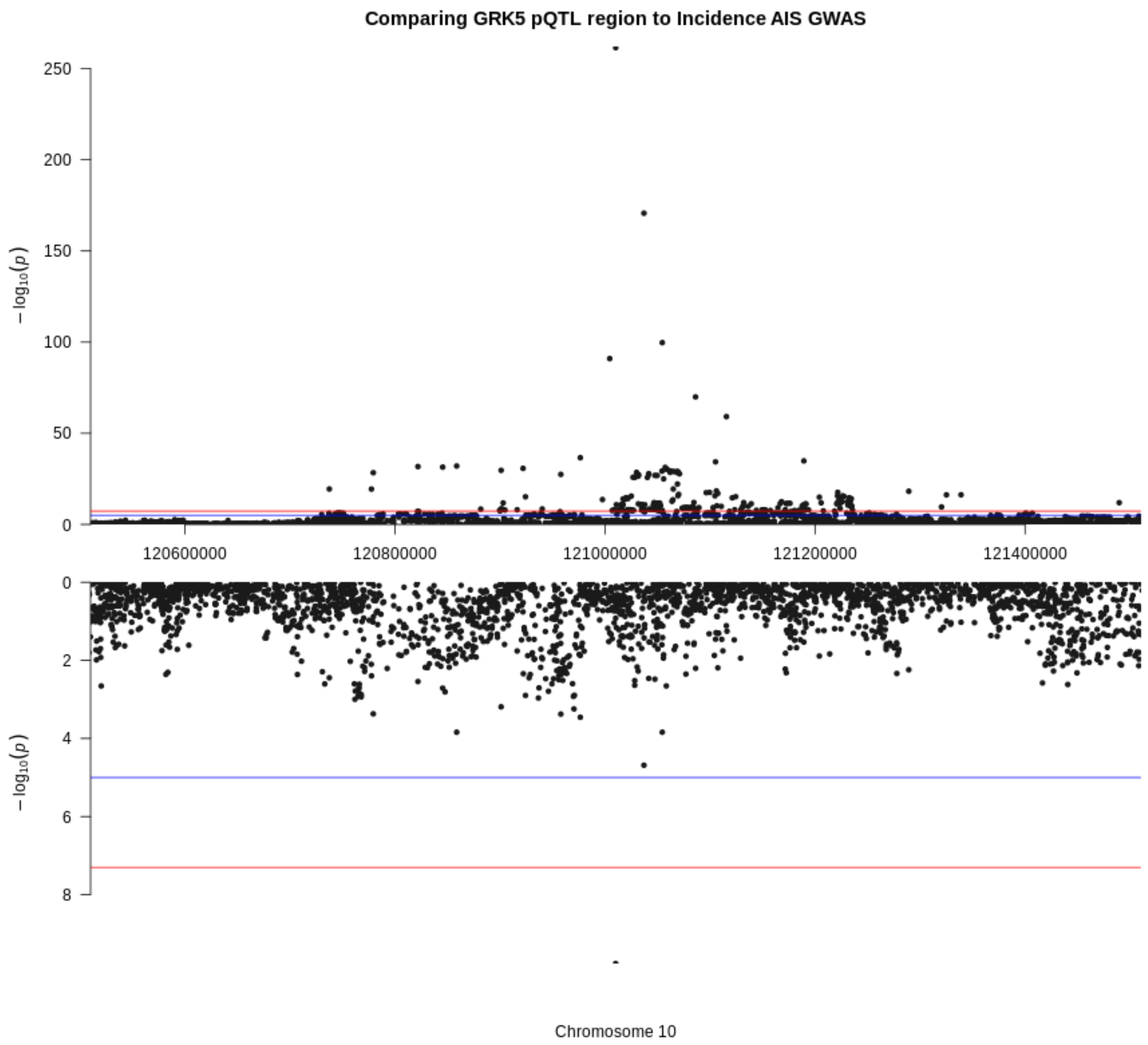

Comparing GRK5 pQTL region to Incidence AIS GWAS
250
200
150
100
50
0
− log10(p)
120600000
120800000
121000000
121200000
121400000
0
2
4
6
8
− log10(p)
Chromosome 10

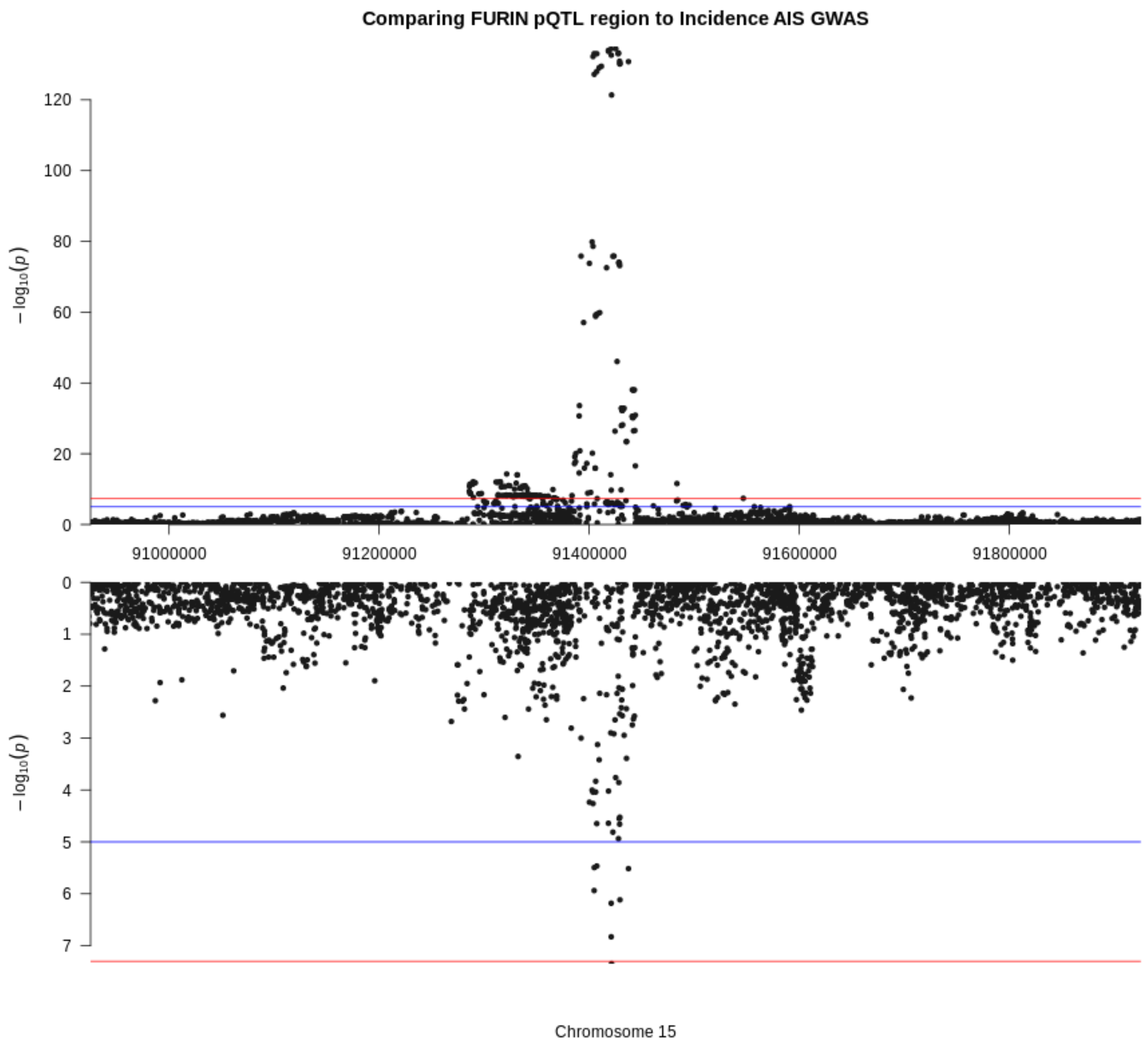
Comparing FURIN pQTL region to Incidence AIS GWAS
−log10(p)
120
100
80
60
40
20
0
91000000
91200000
91400000
91600000
91800000
0
1
2
3
4
5
6
7
−log10(p)
Chromosome 15

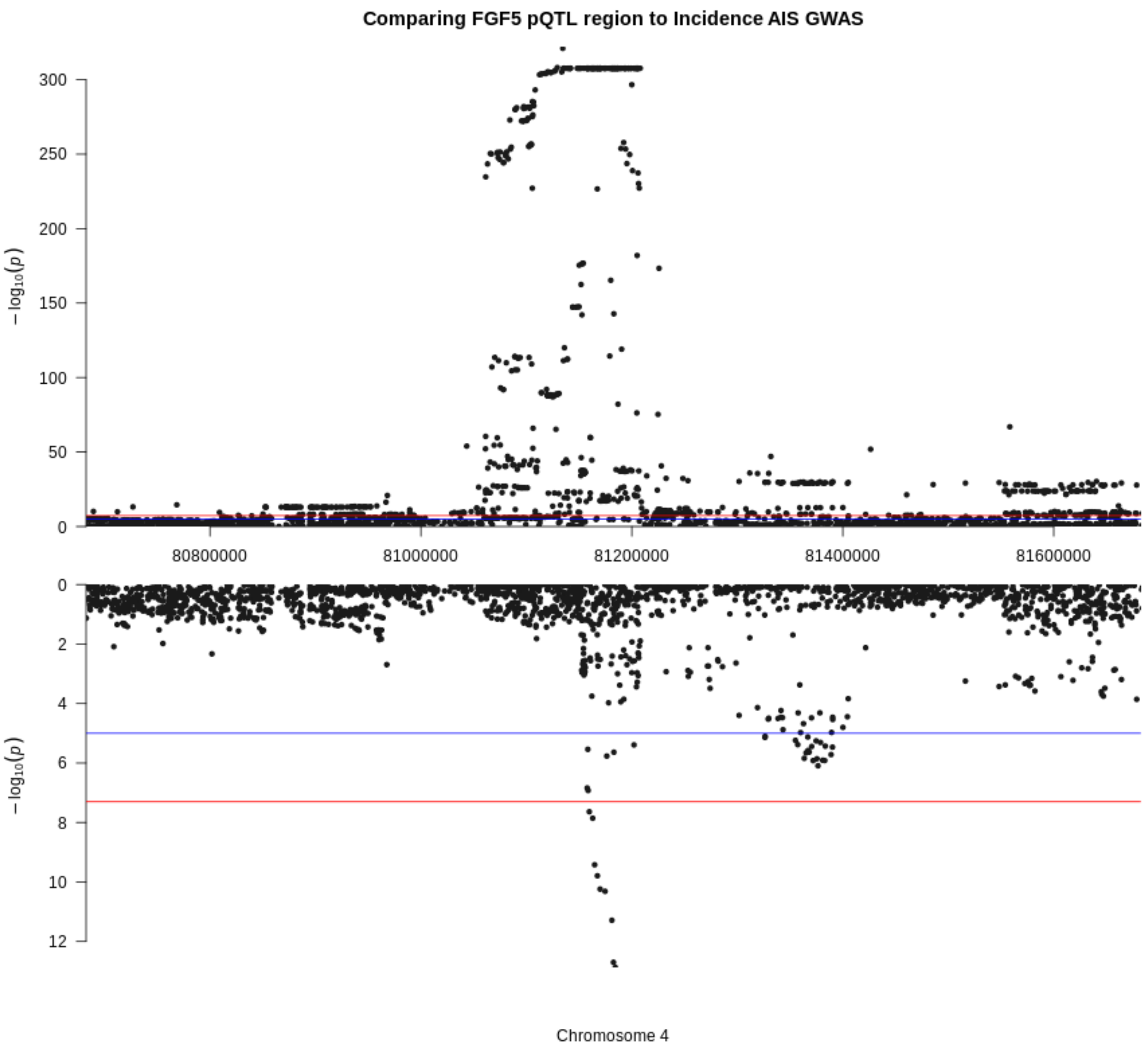
Comparing FGF5 pQTL region to Incidence AIS GWAS
300
250
200
150
100
50
0
−log10(p)
80800000
81000000
81200000
81400000
81600000
0
2
4
6
8
10
12
−log10(p)
Chromosome 4

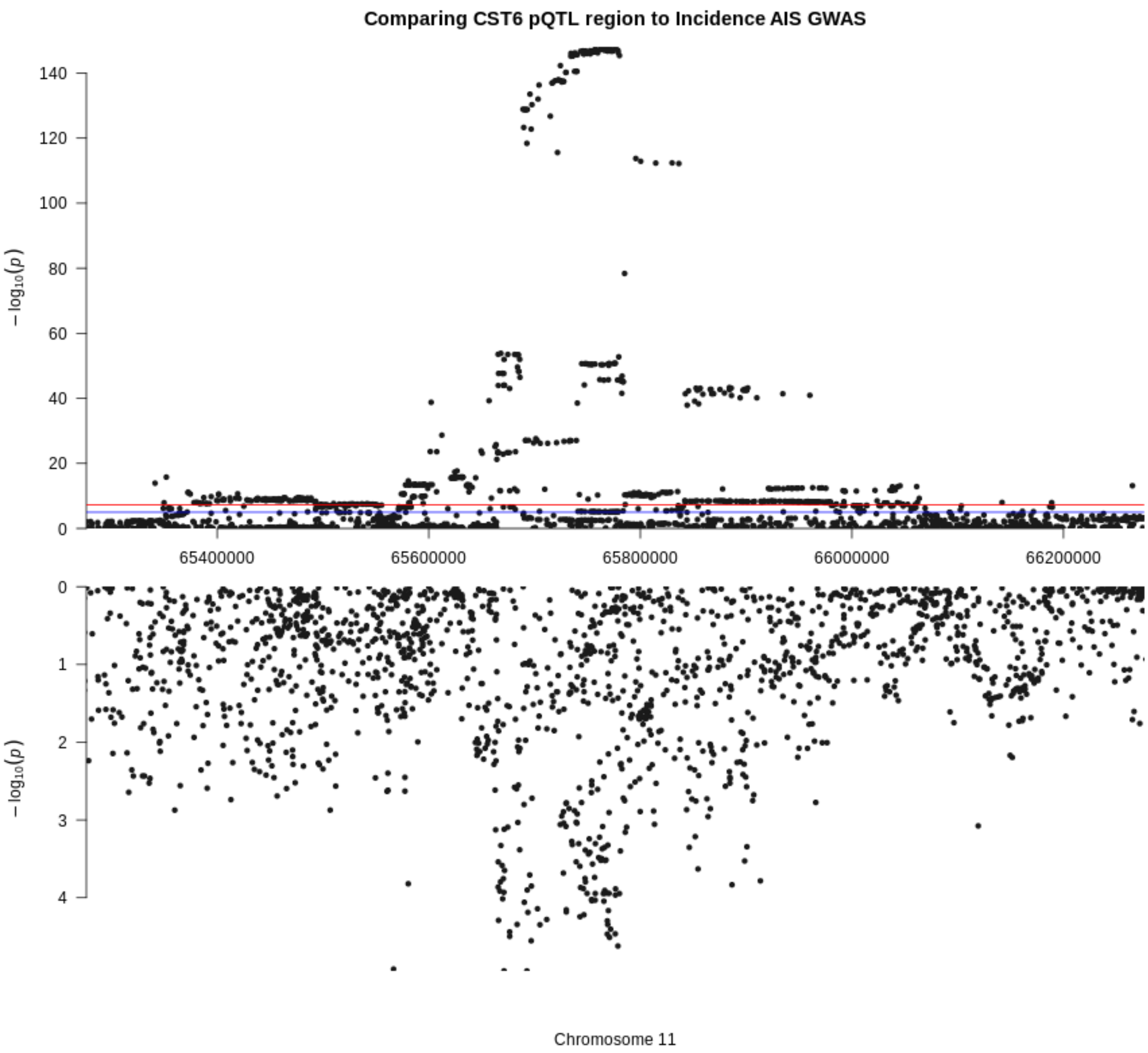
Comparing CST6 pQTL region to Incidence AIS GWAS
140
120
100
80
60
40
20
0
− log10(p)
65400000
65600000
65800000
66000000
66200000
0
1
2
3
4
− log10(p)
Chromosome 11

Figure S6: Miami Plots Comparing pQTL data and Subsequent MACE GWAS (both unadjusted and Slope-Hunter adjusted) in areas highlighted by MR Results

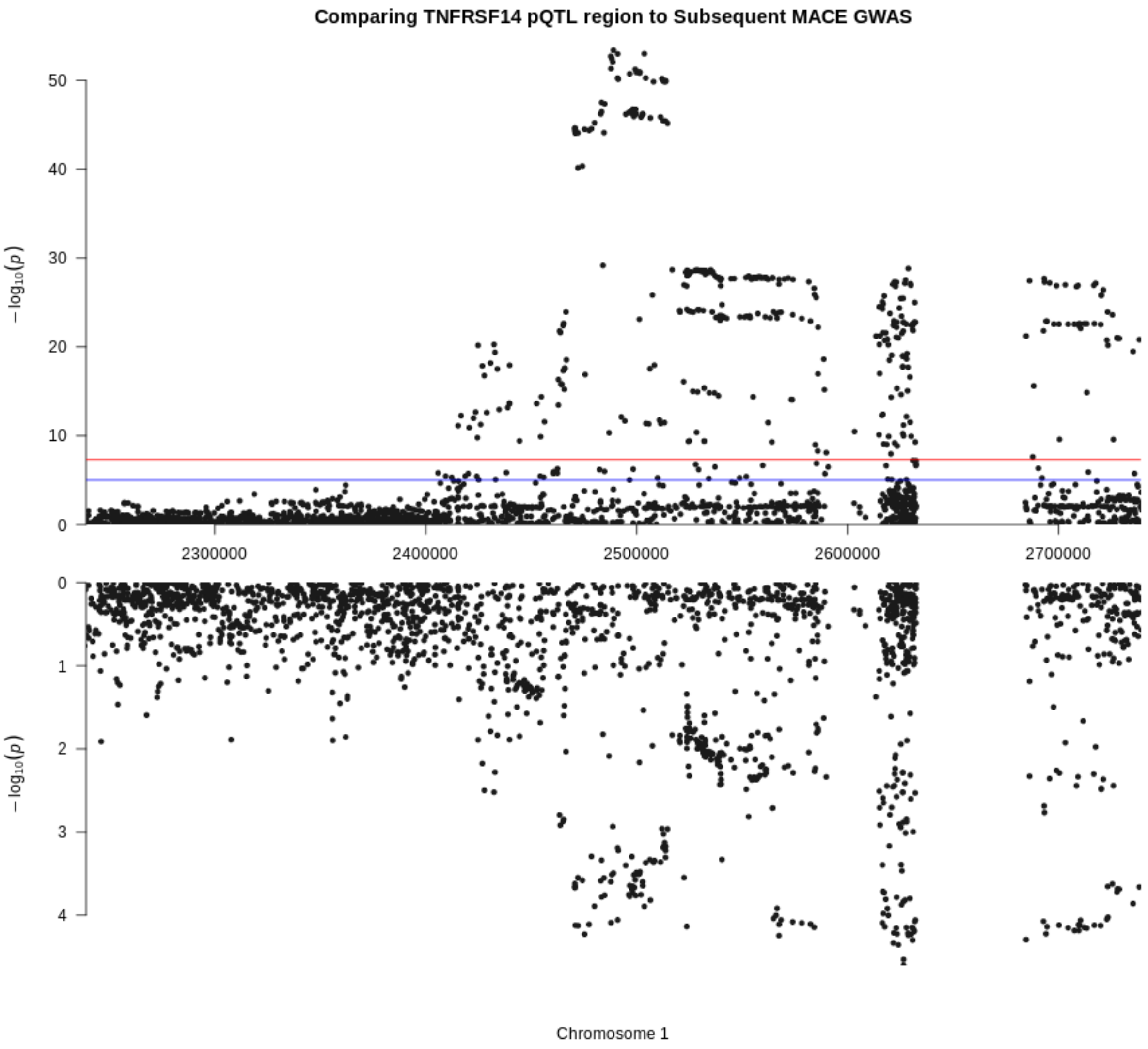

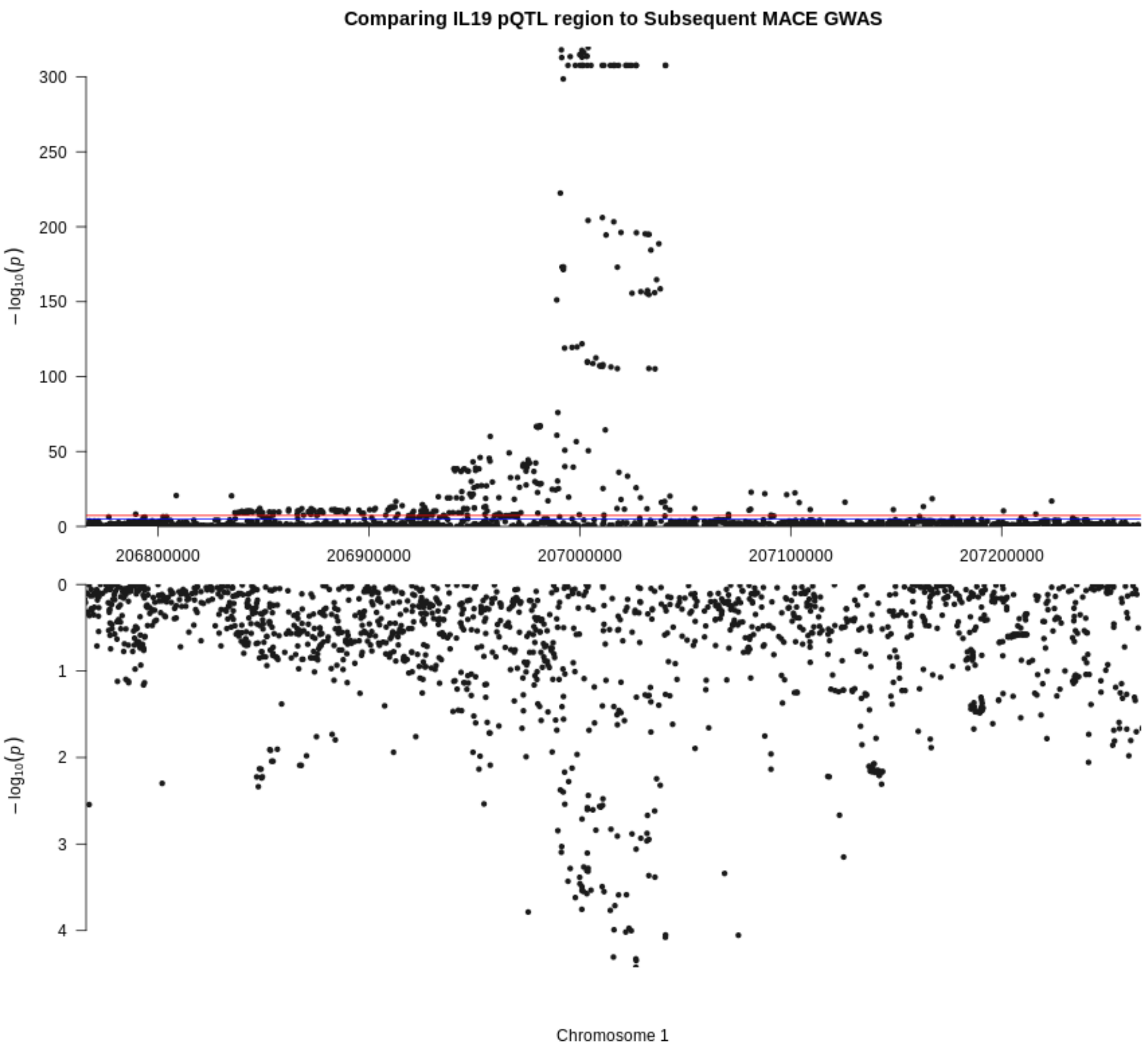
Comparing IL19 pQTL region to Subsequent MACE GWAS
300
250
200
150
100
50
0
$-\log_{10}(p)$
206800000
206900000
207000000
207100000
207200000
0
1
2
3
4
$-\log_{10}(p)$
Chromosome 1

Comparing CCL27 pQTL region to Subsequent MACE GWAS

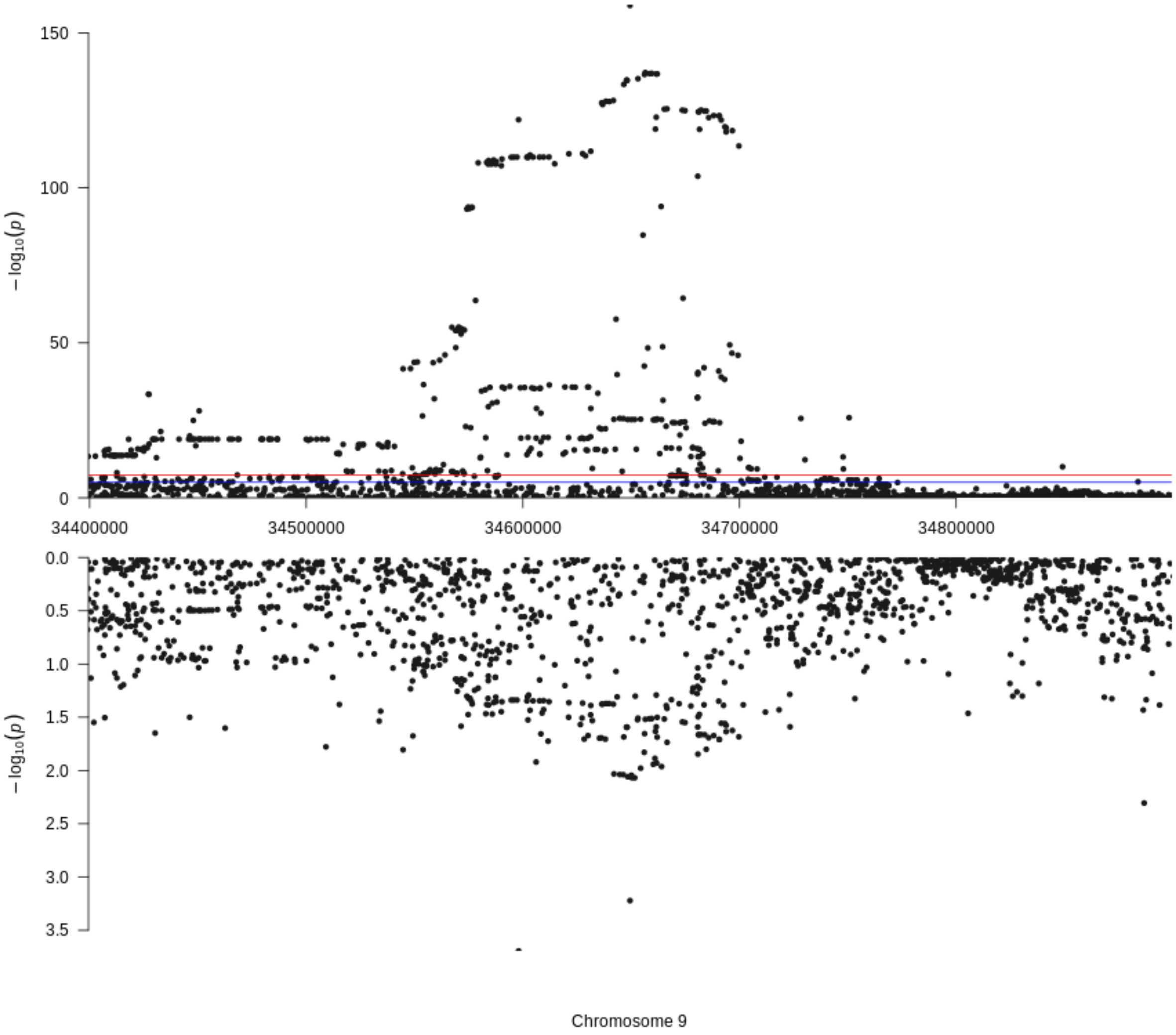

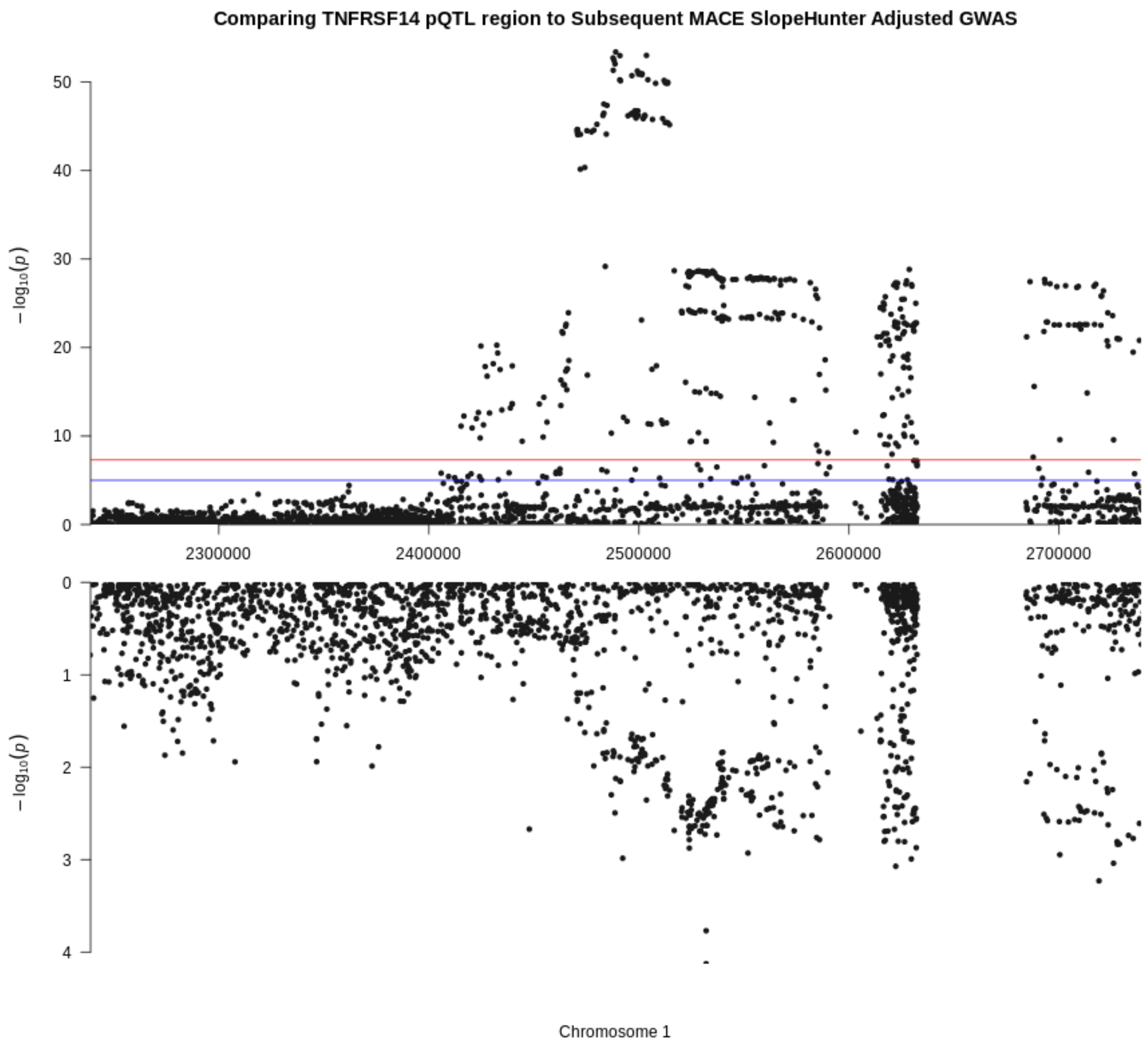

Comparing TNFRSF14 pQTL region to Subsequent MACE SlopeHunter Adjusted GWAS
−log10(p)
50
40
30
20
10
0
2300000
2400000
2500000
2600000
2700000
0
1
2
3
4
Chromosome 1

Comparing IL19 pQTL region to Subsequent MACE SlopeHunter Adjusted GWAS

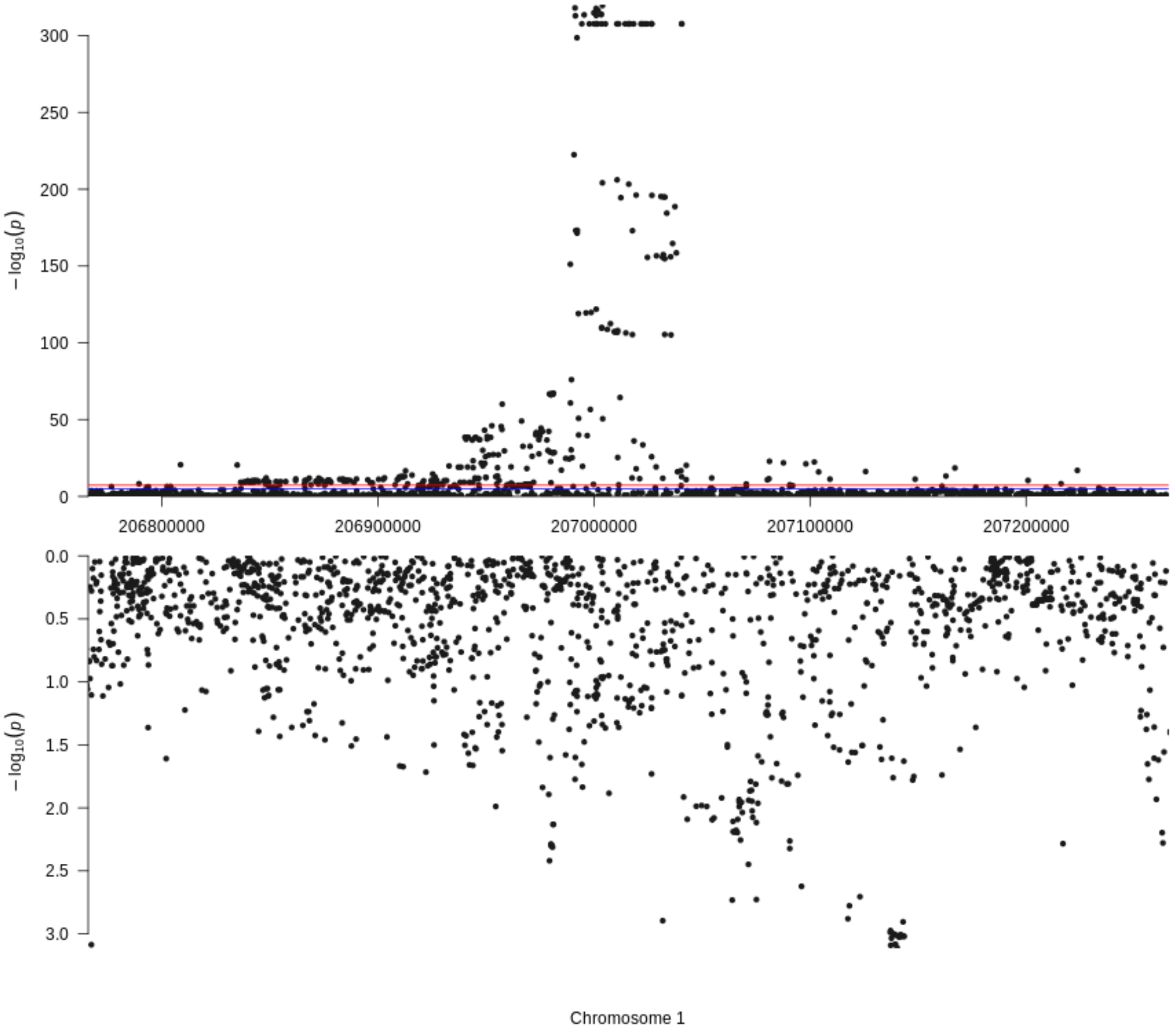

Comparing CCL27 pQTL region to Subsequent MACE SlopeHunter Adjusted GWAS

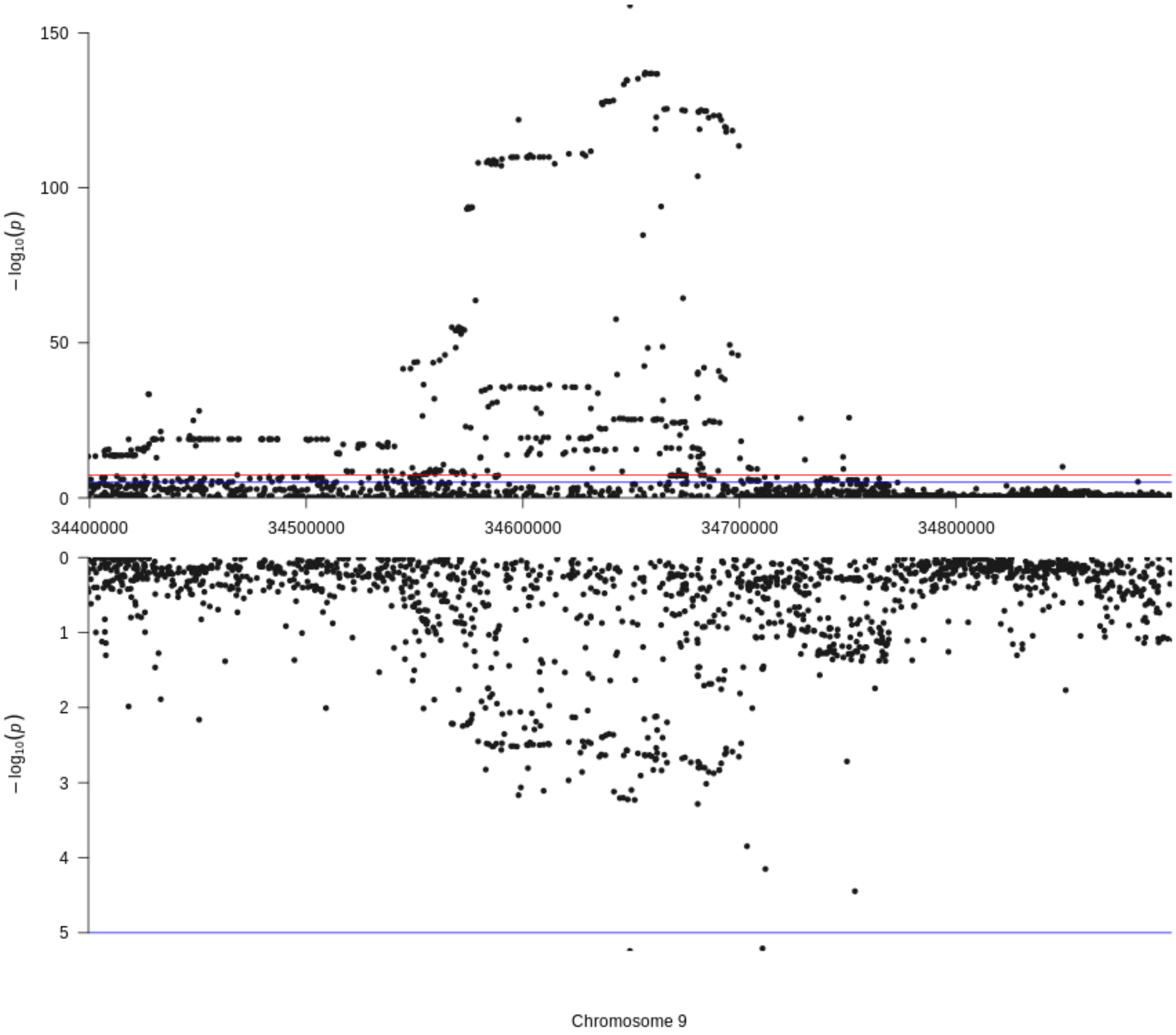

Figure S7: Miami Plots Comparing pQTL data and Subsequent AIS GWAS (both unadjusted and Slope-Hunter adjusted) in areas highlighted by MR Results

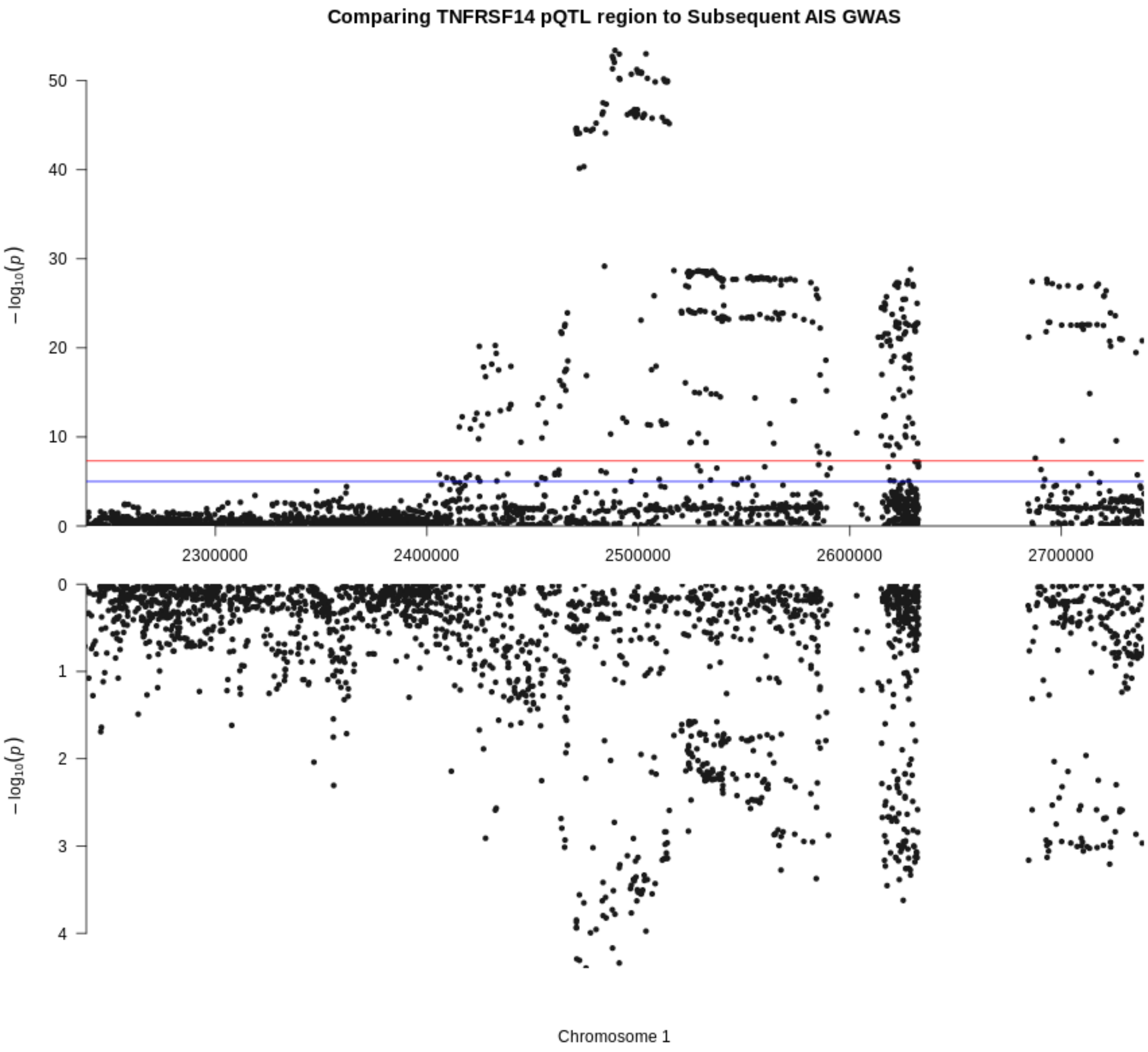

Comparing IL19 pQTL region to Subsequent AIS GWAS

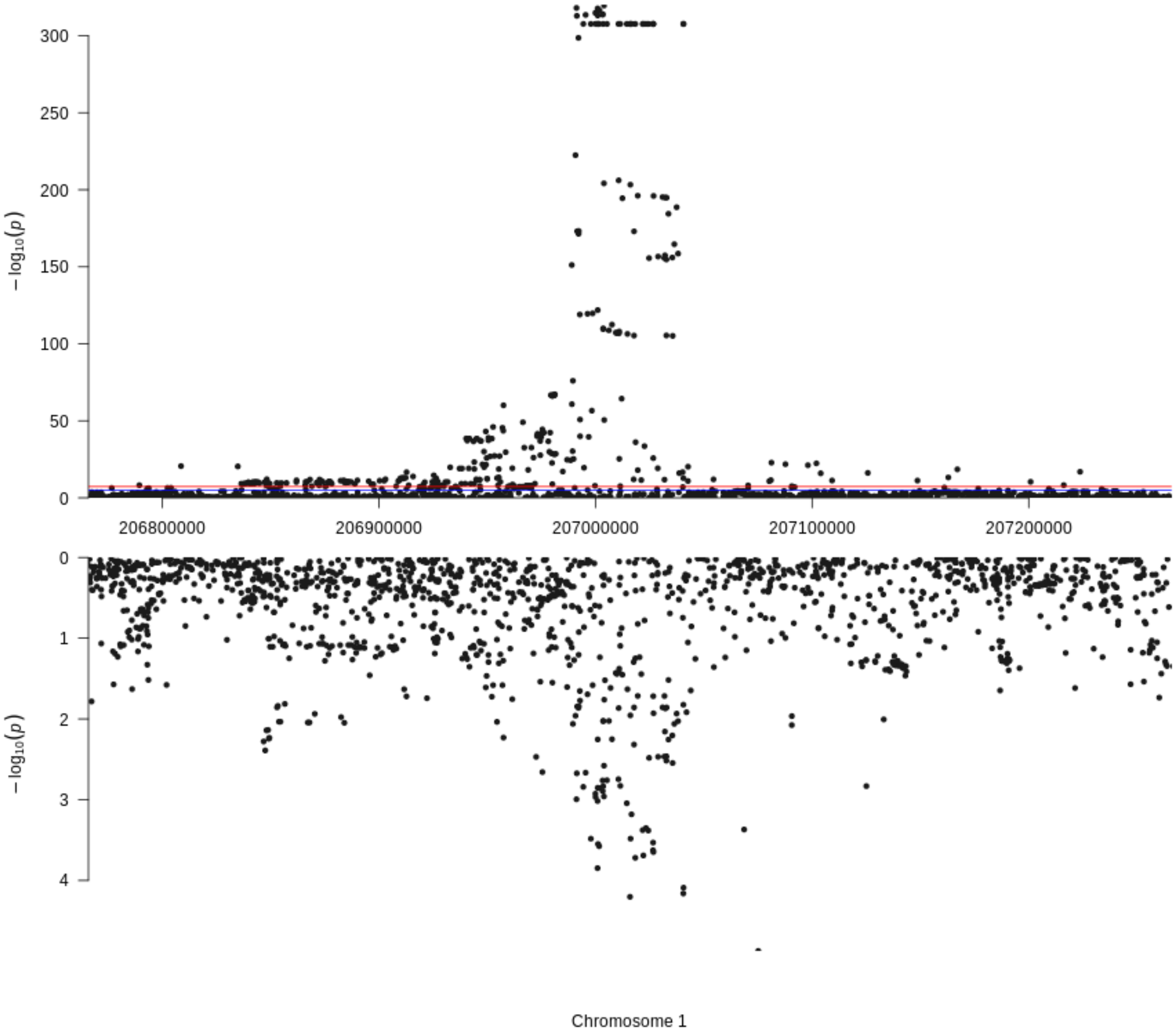

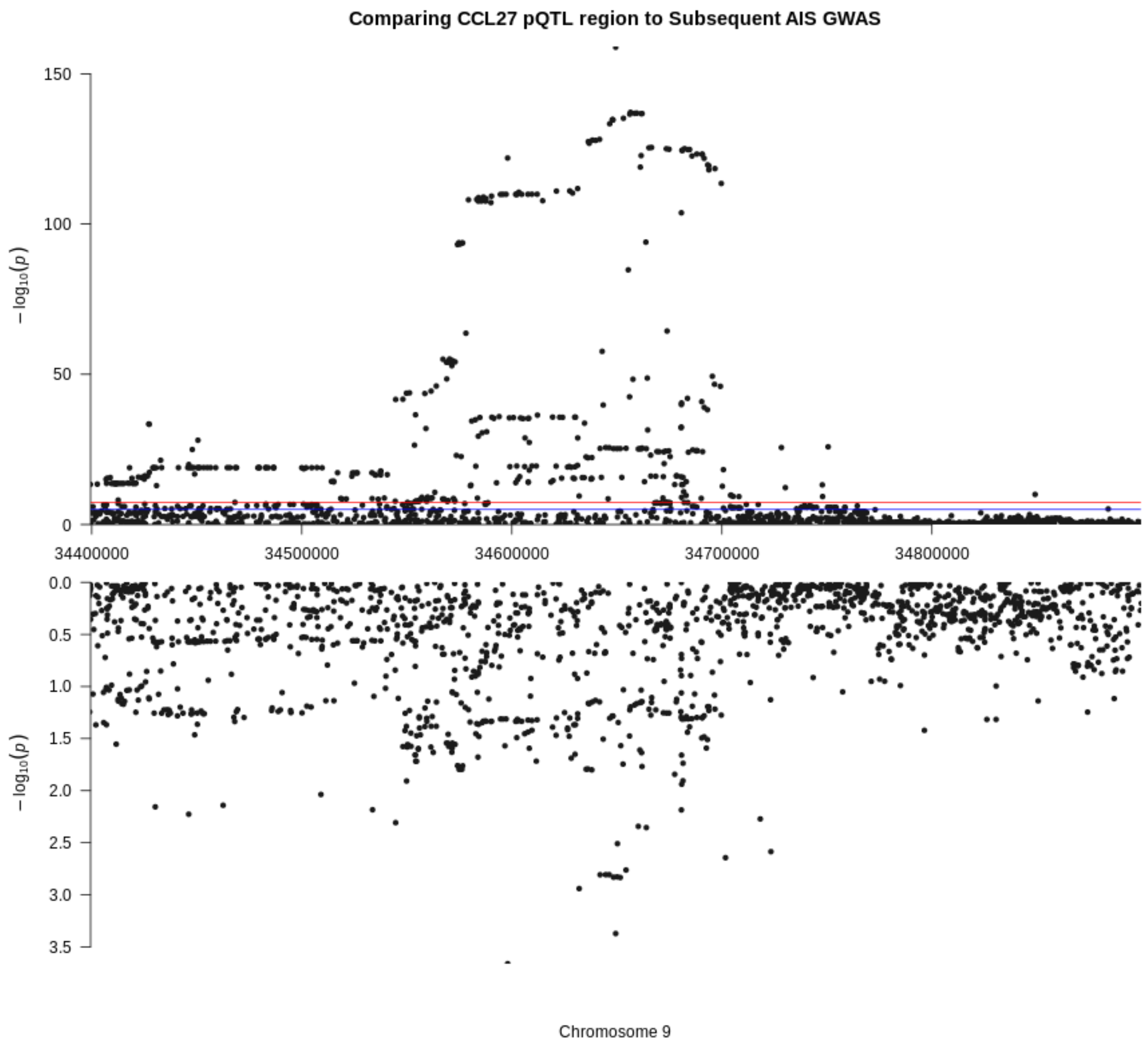
Comparing CCL27 pQTL region to Subsequent AIS GWAS
150
100
50
0
−log10(p)
34400000
34500000
34600000
34700000
34800000
0.0
0.5
1.0
1.5
2.0
2.5
3.0
3.5
−log10(p)
Chromosome 9

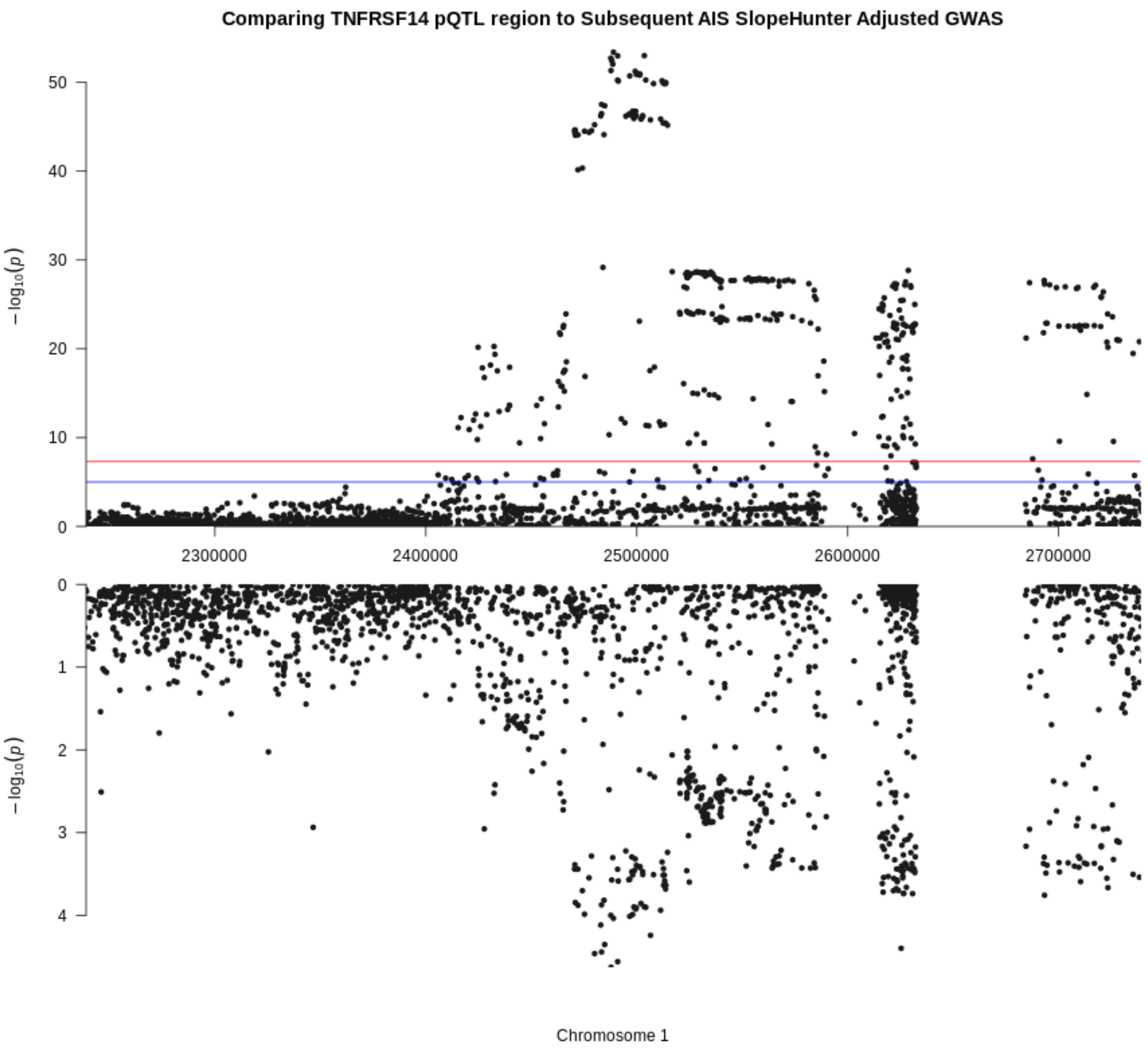

Comparing TNFRSF14 pQTL region to Subsequent AIS SlopeHunter Adjusted GWAS
50
40
30
20
10
0
2300000
2400000
2500000
2600000
2700000
1
2
3
4
Chromosome 1

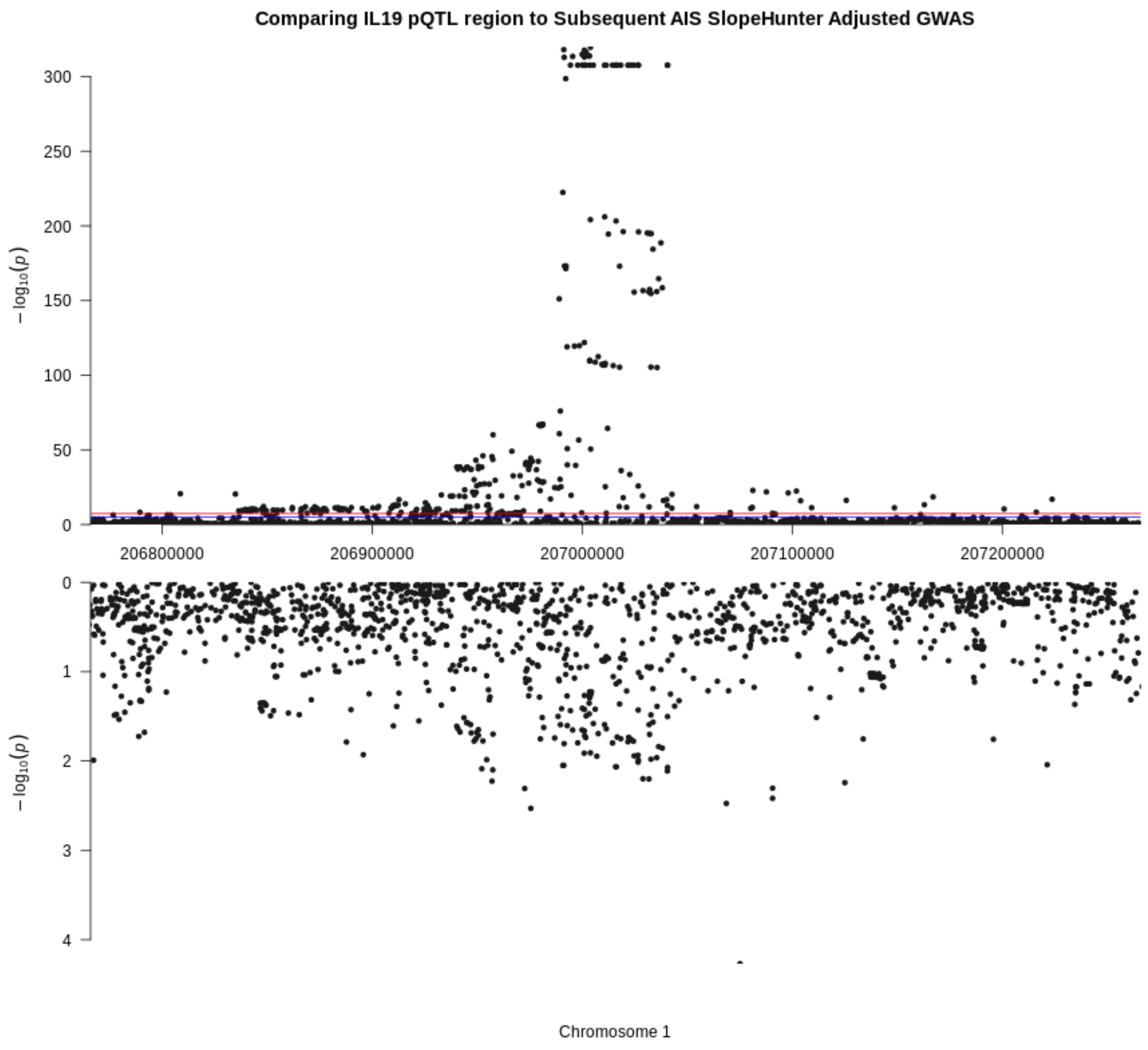

Comparing IL19 pQTL region to Subsequent AIS SlopeHunter Adjusted GWAS
300
250
200
150
100
50
0
$-\log_{10}(p)$
206800000
206900000
207000000
207100000
207200000
0
1
2
3
4
$-\log_{10}(p)$
Chromosome 1

Comparing CCL27 pQTL region to Subsequent AIS SlopeHunter Adjusted GWAS

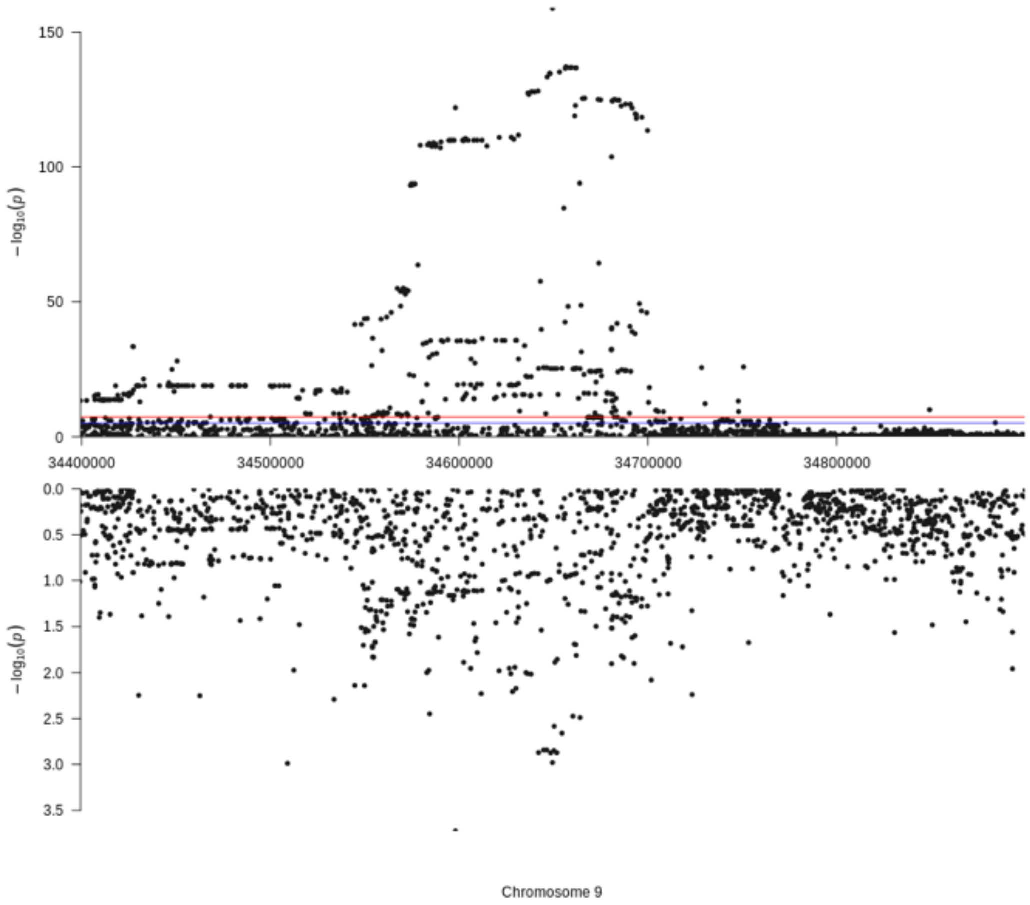

VA Million Veteran Program:
Core Acknowledgement for Publications
February 2023

MVP Program Office

- Sumitra Muralidhar, Ph.D., Program Director
  US Department of Veterans Affairs, 810 Vermont Avenue NW, Washington, DC 20420
- Jennifer Moser, Ph.D., Associate Director, Scientific Programs
  US Department of Veterans Affairs, 810 Vermont Avenue NW, Washington, DC 20420
- Jennifer E. Deen, B.S., Associate Director, Cohort & Public Relations
  US Department of Veterans Affairs, 810 Vermont Avenue NW, Washington, DC 20420

MVP Executive Committee

- Co-Chair: Philip S. Tsao, Ph.D.
  VA Palo Alto Health Care System, 3801 Miranda Avenue, Palo Alto, CA 94304
- Co-Chair: Sumitra Muralidhar, Ph.D.
  US Department of Veterans Affairs, 810 Vermont Avenue NW, Washington, DC 20420
- J. Michael Gaziano, M.D., M.P.H.
  VA Boston Healthcare System, 150 S. Huntington Avenue, Boston, MA 02130
- Elizabeth Hauser, Ph.D.
  Durham VA Medical Center, 508 Fulton Street, Durham, NC 27705
- Amy Kilbourne, Ph.D., M.P.H.
  VA HSR&D, 2215 Fuller Road, Ann Arbor, MI 48105
- Shiuh-Wen Luoh, M.D., Ph.D.
  VA Portland Health Care System, 3710 SW US Veterans Hospital Rd, Portland, OR 97239
- Michael Matheny, M.D., M.S., M.P.H.
  VA Tennessee Valley Healthcare System, 1310 24th Ave. South, Nashville, TN 37212
- Dave Oslin, M.D.
  Philadelphia VA Medical Center, 3900 Woodland Avenue, Philadelphia, PA 19104

MVP Co-Principal Investigators

- J. Michael Gaziano, M.D., M.P.H.
  VA Boston Healthcare System, 150 S. Huntington Avenue, Boston, MA 02130
- Philip S. Tsao, Ph.D.
  VA Palo Alto Health Care System, 3801 Miranda Avenue, Palo Alto, CA 94304

MVP Core Operations

- Lori Churby, B.S., Director, MVP Regulatory Affairs
  VA Palo Alto Health Care System, 3801 Miranda Avenue, Palo Alto, CA 94304
- Stacey B. Whitbourne, Ph.D., Director, MVP Cohort Management

VA Boston Healthcare System, 150 S. Huntington Avenue, Boston, MA 02130

- Jessica V. Brewer, M.P.H., Director, MVP Recruitment & Enrollment
  VA Boston Healthcare System, 150 S. Huntington Avenue, Boston, MA 02130
- Shahpoor (Alex) Shayan, M.S., Director, MVP Recruitment and Enrollment Informatics
  VA Boston Healthcare System, 150 S. Huntington Avenue, Boston, MA 02130
- Luis E. Selva, Ph.D., Executive Director, MVP Biorepositories
  VA Boston Healthcare System, 150 S. Huntington Avenue, Boston, MA 02130
- Saiju Pyarajan Ph.D., Director, Data and Computational Sciences
  VA Boston Healthcare System, 150 S. Huntington Avenue, Boston, MA 02130
- Kelly Cho, M.P.H, Ph.D., Director, MVP Phenomics Data Core
  VA Boston Healthcare System, 150 S. Huntington Avenue, Boston, MA 02130
- Scott L. DuVall, Ph.D., Director, VA Informatics and Computing Infrastructure (VINCI)
  VA Salt Lake City Health Care System, 500 Foothill Drive, Salt Lake City, UT 84148
- Mary T. Brophy M.D., M.P.H., Director, VA Central Biorepository
  VA Boston Healthcare System, 150 S. Huntington Avenue, Boston, MA 02130
- MVP Coordinating Centers
  - MVP Coordinating Center, Boston - J. Michael Gaziano, M.D., M.P.H.
    VA Boston Healthcare System, 150 S. Huntington Avenue, Boston, MA 02130
  - MVP Coordinating Center, Palo Alto – Philip S. Tsao, Ph.D.
    VA Palo Alto Health Care System, 3801 Miranda Avenue, Palo Alto, CA 94304
  - MVP Information Center, Canandaigua – Brady Stephens, M.S.
    Canandaigua VA Medical Center, 400 Fort Hill Avenue, Canandaigua, NY 14424
  - Cooperative Studies Program Clinical Research Pharmacy Coordinating Center, Albuquerque – Todd Connor, Pharm.D.; Dean P. Argyres, B.S., M.S.
    New Mexico VA Health Care System, 1501 San Pedro Drive SE, Albuquerque, NM 87108

MVP Publications and Presentations Committee

- Co-Chair: Themistocles L. Assimes, M.D., Ph. D
  VA Palo Alto Health Care System, 3801 Miranda Avenue, Palo Alto, CA 94304
- Co-Chair: Adriana Hung, M.D.; M.P.H
  VA Tennessee Valley Healthcare System, 1310 24th Ave. South, Nashville, TN 37212
- Co-Chair: Henry Kranzler, M.D.
  Philadelphia VA Medical Center, 3900 Woodland Avenue, Philadelphia, PA 19104

MVP Local Site Investigators

- Samuel Aguayo, M.D., Phoenix VA Health Care System
  650 E. Indian School Road, Phoenix, AZ 85012
- Sunil Ahuja, M.D., South Texas Veterans Health Care System
  7400 Merton Minter Boulevard, San Antonio, TX 78229
- Kathrina Alexander, M.D., Veterans Health Care System of the Ozarks
  1100 North College Avenue, Fayetteville, AR 72703

- Xiao M. Androulakis, M.D., Columbia VA Health Care System
  6439 Garners Ferry Road, Columbia, SC 29209
- Prakash Balasubramanian, M.D., William S. Middleton Memorial Veterans Hospital
  2500 Overlook Terrace, Madison, WI 53705
- Zuhair Ballas, M.D., Iowa City VA Health Care System
  601 Highway 6 West, Iowa City, IA 52246-2208
- Jean Beckham, Ph.D., Durham VA Medical Center
  508 Fulton Street, Durham, NC 27705
- Sujata Bhushan, M.D., VA North Texas Health Care System
  4500 S. Lancaster Road, Dallas, TX 75216
- Edward Boyko, M.D., VA Puget Sound Health Care System
  1660 S. Columbian Way, Seattle, WA 98108-1597
- David Cohen, M.D., Portland VA Medical Center
  3710 SW U.S. Veterans Hospital Road, Portland, OR 97239
- Louis Dellitalia, M.D., Birmingham VA Medical Center
  700 S. 19th Street, Birmingham AL 35233
- L. Christine Faulk, M.D., Robert J. Dole VA Medical Center
  5500 East Kellogg Drive, Wichita, KS 67218-1607
- Joseph Fayad, M.D., VA Southern Nevada Healthcare System
  6900 North Pecos Road, North Las Vegas, NV 89086
- Daryl Fujii, Ph.D., VA Pacific Islands Health Care System
  459 Patterson Rd, Honolulu, HI 96819
- Saib Gappy, M.D., John D. Dingell VA Medical Center
  4646 John R Street, Detroit, MI 48201
- Frank Gesek, Ph.D., White River Junction VA Medical Center
  163 Veterans Drive, White River Junction, VT 05009
- Jennifer Greco, M.D., Sioux Falls VA Health Care System
  2501 W 22nd Street, Sioux Falls, SD 57105
- Michael Godschalk, M.D., Richmond VA Medical Center
  1201 Broad Rock Blvd., Richmond, VA 23249
- Todd W. Gress, M.D., Ph.D., Hershel “Woody” Williams VA Medical Center
  1540 Spring Valley Drive, Huntington, WV 25704
- Samir Gupta, M.D., M.S.C.S., VA San Diego Healthcare System
  3350 La Jolla Village Drive, San Diego, CA 92161
- Salvador Gutierrez, M.D., Edward Hines, Jr. VA Medical Center
  5000 South 5th Avenue, Hines, IL 60141
- John Harley, M.D., Ph.D., Cincinnati VA Medical Center
  3200 Vine Street, Cincinnati, OH 45220
- Kimberly Hammer, Ph.D., Fargo VA Health Care System
  2101 N. Elm, Fargo, ND 58102
- Mark Hamner, M.D., Ralph H. Johnson VA Medical Center
  109 Bee Street, Mental Health Research, Charleston, SC 29401
- Adriana Hung, M.D., M.P.H., VA Tennessee Valley Healthcare System
  1310 24th Avenue, South Nashville, TN 37212

- Robin Hurley, M.D., W.G. (Bill) Hefner VA Medical Center
  1601 Brenner Ave, Salisbury, NC 28144
- Pran Iruvanti, D.O., Ph.D., Hampton VA Medical Center
  100 Emancipation Drive, Hampton, VA 23667
- Frank Jacono, M.D., VA Northeast Ohio Healthcare System
  10701 East Boulevard, Cleveland, OH 44106
- Darshana Jhala, M.D., Philadelphia VA Medical Center
  3900 Woodland Avenue, Philadelphia, PA 19104
- Scott Kinlay, M.B.B.S., Ph.D., VA Boston Healthcare System
  150 S. Huntington Avenue, Boston, MA 02130
- Jon Klein, M.D., Ph.D., Louisville VA Medical Center
  800 Zorn Avenue, Louisville, KY 40206
- Michael Landry, Ph.D., Southeast Louisiana Veterans Health Care System
  2400 Canal Street, New Orleans, LA 70119
- Peter Liang, M.D., M.P.H., VA New York Harbor Healthcare System
  423 East 23rd Street, New York, NY 10010
- Suthat Liangpunsakul, M.D., M.P.H., Richard Roudebush VA Medical Center
  1481 West 10th Street, Indianapolis, IN 46202
- Jack Lichy, M.D., Ph.D., Washington DC VA Medical Center
  50 Irving St, Washington, D. C. 20422
- C. Scott Mahan, M.D., Charles George VA Medical Center
  1100 Tunnel Road, Asheville, NC 28805
- Ronnie Marrache, M.D., VA Maine Healthcare System
  1 VA Center, Augusta, ME 04330
- Stephen Mastorides, M.D., James A. Haley Veterans' Hospital
  13000 Bruce B. Downs Blvd, Tampa, FL 33612
- Elisabeth Mates M.D., Ph.D., VA Sierra Nevada Health Care System
  975 Kirman Avenue, Reno, NV 89502
- Kristin Mattocks, Ph.D., M.P.H., Central Western Massachusetts Healthcare System
  421 North Main Street, Leeds, MA 01053
- Paul Meyer, M.D., Ph.D., Southern Arizona VA Health Care System
  3601 S 6th Avenue, Tucson, AZ 85723
- Jonathan Moorman, M.D., Ph.D., James H. Quillen VA Medical Center
  Corner of Lamont & Veterans Way, Mountain Home, TN 37684
- Timothy Morgan, M.D., VA Long Beach Healthcare System
  5901 East 7th Street Long Beach, CA 90822
- Maureen Murdoch, M.D., M.P.H., Minneapolis VA Health Care System
  One Veterans Drive, Minneapolis, MN 55417
- James Norton, Ph.D., VA Health Care Upstate New York
  113 Holland Avenue, Albany, NY 12208
- Olaoluwa Okusaga, M.D., Michael E. DeBakey VA Medical Center
  2002 Holcombe Blvd, Houston, TX 77030
- Kris Ann Oursler, M.D., Salem VA Medical Center
  1970 Roanoke Blvd, Salem, VA 24153

- Ana Palacio, M.D., M.P.H., Miami VA Health Care System
  1201 NW 16th Street, 11 GRC, Miami FL 33125
- Samuel Poon, M.D., Manchester VA Medical Center
  718 Smyth Road, Manchester, NH 03104
- Emily Potter, Pharm.D., VA Eastern Kansas Health Care System
  4101 S 4th Street Trafficway, Leavenworth, KS 66048
- Michael Rauchman, M.D., St. Louis VA Health Care System
  915 North Grand Blvd, St. Louis, MO 63106
- Richard Servatius, Ph.D., Syracuse VA Medical Center
  800 Irving Avenue, Syracuse, NY 13210
- Satish Sharma, M.D., Providence VA Medical Center
  830 Chalkstone Avenue, Providence, RI 02908
- River Smith, Ph.D., Eastern Oklahoma VA Health Care System
  1011 Honor Heights Drive, Muskogee, OK 74401
- Peruvemba Sriram, M.D., N. FL/S. GA Veterans Health System
  1601 SW Archer Road, Gainesville, FL 32608
- Patrick Strollo, Jr., M.D., VA Pittsburgh Health Care System
  University Drive, Pittsburgh, PA 15240
- Neeraj Tandon, M.D., Overton Brooks VA Medical Center
  510 East Stoner Ave, Shreveport, LA 71101
- Philip Tsao, Ph.D., VA Palo Alto Health Care System
  3801 Miranda Avenue, Palo Alto, CA 94304-1290
- Gerardo Villareal, M.D., New Mexico VA Health Care System
  1501 San Pedro Drive, S.E. Albuquerque, NM 87108
- Agnes Wallbom, M.D., M.S., VA Greater Los Angeles Health Care System
  11301 Wilshire Blvd, Los Angeles, CA 90073
- Jessica Walsh, M.D., VA Salt Lake City Health Care System
  500 Foothill Drive, Salt Lake City, UT 84148
- John Wells, Ph.D., Edith Nourse Rogers Memorial Veterans Hospital
  200 Springs Road, Bedford, MA 01730
- Jeffrey Whittle, M.D., M.P.H., Clement J. Zablocki VA Medical Center
  5000 West National Avenue, Milwaukee, WI 53295
- Mary Whooley, M.D., San Francisco VA Health Care System
  4150 Clement Street, San Francisco, CA 94121
- Allison E. Williams, N.D., Ph.D., R.N, Bay Pines VA Healthcare System
  10,000 Bay Pines Blvd Bay Pines, FL 33744
- Peter Wilson, M.D., Atlanta VA Medical Center
  1670 Clairmont Road, Decatur, GA 30033
- Junzhe Xu, M.D., VA Western New York Healthcare System
  3495 Bailey Avenue, Buffalo, NY 14215-1199
- Shing Shing Yeh, Ph.D., M.D., Northport VA Medical Center
  79 Middleville Road, Northport, NY 11768
